# Mitochondrial hypoxic stress induces widespread RNA editing by APOBEC3G in natural killer cells

**DOI:** 10.1186/s13059-019-1651-1

**Published:** 2019-02-21

**Authors:** Shraddha Sharma, Jianmin Wang, Emad Alqassim, Scott Portwood, Eduardo Cortes Gomez, Orla Maguire, Per H. Basse, Eunice S. Wang, Brahm H. Segal, Bora E. Baysal

**Affiliations:** 1 Department of Pathology and Laboratory Medicine, Roswell Park Comprehensive Cancer Center, Buffalo, NY 14263 USA; 2 Department of Bioinformatics and Biostatistics, Roswell Park Comprehensive Cancer Center, Buffalo, NY 14263 USA; 3 Department of Internal Medicine, Roswell Park Comprehensive Cancer Center, Buffalo, NY 14263 USA; 4 Department of Medicine, Roswell Park Comprehensive Cancer Center, Buffalo, NY 14263 USA; 5 Department of Flow and Image Cytometry, Roswell Park Comprehensive Cancer Center, Buffalo, NY 14263 USA; 6 Present Address: Translate Bio, Lexington, MA 02421 USA

**Keywords:** RNA editing, APOBEC3, NK cells, Hypoxia, Cell stress, Mitochondria, Epitranscriptome, Innate immune cells, Gene knockdown

## Abstract

**Background:**

Protein recoding by RNA editing is required for normal health and evolutionary adaptation. However, de novo induction of RNA editing in response to environmental factors is an uncommon phenomenon. While APOBEC3A edits many mRNAs in monocytes and macrophages in response to hypoxia and interferons, the physiological significance of such editing is unclear.

**Results:**

Here, we show that the related cytidine deaminase, APOBEC3G, induces site-specific C-to-U RNA editing in natural killer cells, lymphoma cell lines, and, to a lesser extent, CD8-positive T cells upon cellular crowding and hypoxia. In contrast to expectations from its anti-HIV-1 function, the highest expression of APOBEC3G is shown to be in cytotoxic lymphocytes. RNA-seq analysis of natural killer cells subjected to cellular crowding and hypoxia reveals widespread C-to-U mRNA editing that is enriched for genes involved in mRNA translation and ribosome function. APOBEC3G promotes Warburg-like metabolic remodeling in HuT78 T cells under similar conditions. Hypoxia-induced RNA editing by APOBEC3G can be mimicked by the inhibition of mitochondrial respiration and occurs independently of HIF-1α.

**Conclusions:**

APOBEC3G is an endogenous RNA editing enzyme in primary natural killer cells and lymphoma cell lines. This RNA editing is induced by cellular crowding and mitochondrial respiratory inhibition to promote adaptation to hypoxic stress.

**Electronic supplementary material:**

The online version of this article (10.1186/s13059-019-1651-1) contains supplementary material, which is available to authorized users.

## Background

RNA editing is an evolutionarily conserved post-transcriptional modification that can result in amino acid recoding and altered protein function [1]. Protein recoding RNA editing plays an important role during development and in helping organisms adapt to changes in the environment. A-to-I (A>I) and C-to-U (C>U) are the two most common types of RNA editing in mammals, carried out by the ADAR and APOBEC enzymes, respectively.

The more widespread ADAR-mediated A>I RNA editing, mostly occurs (~ 98%) in non-coding repetitive regions [2], likely to combat viral infection and to regulate innate immunity, to prevent retrotransposon insertion in the genome [3] or to affect the RNA processing pathway [4]. Environmental factors such as hypoxia or neural activity can modify the level of A>I editing in RNAs of certain genes [5, 6], which are already edited under normal physiological conditions (baseline). Recent studies suggest that the evolutionary acquisition of A>I RNA editing sites can facilitate temperature adaptation in octopus, flies, and single-cell organisms [7–10]. However, whether or not RNA editing can be dynamically induced at specific sites de novo in response to environmental factors, especially in mammals, is not understood well.

In mammals, C>U RNA editing by cytidine deamination is infrequent in baseline transcript sequences under normal physiological conditions. An exception is APOBEC1-mediated RNA editing, which is mainly involved in the production of short isoform of the ApoB protein in intestinal cells [11]. The related APOBEC3 (A3) family of enzymes [12, 13], consisting of A3A, A3B, A3C, A3D, A3F, A3G, and A3H, are widely considered as antiviral innate restriction factors because they can mutate foreign genetic material (mainly ssDNA) and inhibit their replication in in vitro models [14]. Recently, we described that APOBEC3A (A3A) induces widespread RNA editing resulting in protein recoding of dozens of genes in primary monocytes when cultured at a high density under hypoxia (low oxygen) or when exposed to interferons (IFN-1 and IFN-γ) and in M1 type macrophages as a result of IFN-γ treatment [15]. However, the relationship between viral restriction and cellular RNA editing by A3A, and the functional significance of such editing, is unknown.

APOBEC3G (A3G), the most studied member of the A3 family, incorporates into vif-deficient HIV-1 virions and inhibits HIV-1 replication in target cells by causing crippling C>U mutations in its minus ssDNA strand and by inhibiting reverse transcription [14, 16]. Interestingly, we found that exogenous transient expression of A3G in HEK293T cells causes C>U editing in mRNAs of hundreds of genes, which are largely distinct from those edited by A3A [17, 18]. A3G has a preference for CC nucleotides both in its ssDNA and RNA substrates, whereas other A3 enzymes prefer TC nucleotides [13, 15, 17–19]. In addition, we find that although RNA editing targets of A3A and A3G are largely distinct, both A3A and A3G prefer to edit Cs located at the 3′-end of tetra- or tri-loops in putative RNA stem-loop structures [15, 17]. While these findings indicate that the A3G enzyme is capable of RNA editing, whether or not such editing occurs endogenously under physiologically relevant conditions is unknown. Therefore, we hypothesized that A3G-mediated RNA editing will be induced in cells which express this enzyme.

In this study, we analyze the cell type-specific expression of A3G and identify widespread RNA editing mediated by A3G, induced by high cell density and hypoxia in natural killer (NK), CD8+ T cells, and in the widely studied Hut78 T cell line. Our findings reveal that under hypoxic stress, A3G-mediated RNA editing converges at targets involved in mRNA translation, likely to reorganize the cellular translation apparatus. Furthermore, we show that A3G promotes adaptation to hypoxic stress by promoting glycolysis over mitochondrial respiration. Thus, A3G is a novel endogenous RNA editing enzyme which can facilitate cellular adaptation to mitochondrial hypoxic cell stress in innate/cytotoxic lymphocytes.

## Results

### Cell type-specific expression of APOBEC3G

**Fig. 1:**
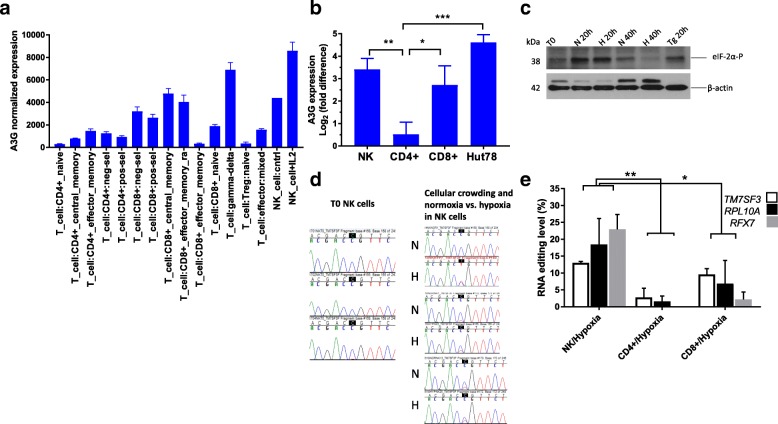
Cell-specific expression of A3G and the induction of RNA editing in NK cells. **a** Cell types that have the highest expression of A3G (probe:204205_at in Affymetrix HG-U133) in Primary Cell Atlas, a meta-analysis of publicly available 100+ microarray datasets, are shown (see Additional file 1; Figure S1 for other immune cells). **b** A3G gene expression in primary NK, CD4+ T, and CD8+ T cells by qPCR (*n* = 3 donors, T0 and hypoxia levels combined, mean and SEM shown). Gene expression measurements are normalized to that of β2-microglobulin. **c** Immunoblot showing the protein levels of eIF-2α phosphorylated at Ser 51 in whole cell lysates of NK cells at 0, 20, and 40 h under normoxia (N) or hypoxia (H). Thapsigargin (Tg)-treated NK cells are used as a positive control, and β-actin is used as a loading control. **d** Sanger sequence chromatogram traces of RT-PCR products of *TM7SF3* in unstressed (uncrowded baseline, T0) and stressed (crowding in normoxia (N) or crowding in hypoxia (H)) NK cells. Edited C is highlighted in black. **e** Estimation of site-specific C>U RNA editing by Sanger sequencing of RT-PCR products for TM7SF3, RPL10A, and RFX7 in NK, CD4+ T, and CD8+ T cells subjected to crowding and hypoxia. (*n* = 3 donors, mean and SEM are shown). See the “Methods” section for statistical analysis

To examine the endogenous RNA editing activity of A3G, we first analyzed A3G’s cell type-specific expression levels. Since RNA editing by A3A is observed in cell types that highly express A3A (monocytes and macrophages), we reasoned that A3G-mediated RNA editing would be more likely to occur in cells that highly express this enzyme. A meta-analysis of the publicly available microarray datasets [20] indicated high expression of A3G in NK cells, gamma delta T cells, and CD8+ T cells (Fig. 1a). Other hematologic and immune cell types have lower A3G expressions (see Additional file 1: Figure S1). Individual gene expression datasets including GeneAtlas U133A [21] and immune-response in silico (IRIS) [22] confirmed a higher expression of A3G in NK cells relative to T and B lymphocytes and myeloid cells. We experimentally confirmed high expression levels of A3G in primary NK and CD8+ T cells but found lower expression in primary CD4+ T cells purified from peripheral blood (Fig. 1b). These results are unexpected because prior studies have implied a potential functional role of A3G in restricting HIV-1 in infected CD4+ T cells [23, 24] whereas other studies did not include NK cells in APOBEC3 gene expression profiling [25, 26]. In contrast, our findings reveal higher expression of A3G in NK and CD8+ T lymphocytes that are not infected by HIV-1.

### Identification of RNA editing by APOBEC3G in NK cells

We have previously shown that A3A, which is highly expressed in monocytes and macrophages, shows very low or the absence of RNA editing activity in these cells when freshly isolated from peripheral blood mononuclear cells (PBMCs) [15]. However, RNA editing is induced when monocytes/macrophages are cultured at a high cell density and low oxygen (hypoxia, 1% O_2_) or by interferons [15, 27]. Since A3G is highly expressed in NK cells, we hypothesized that RNA editing will be induced in NK cells when subjected to hypoxia and/or high cell density. We cultured human PBMCs for 40 h at a high cell density (5 × 10^7^ cells in 1.8 ml per well in a 12-well plate) under normoxia or hypoxia and isolated NK cells. Under these conditions, we observed upregulation of the phosphorylated α subunit of the eukaryotic initiation factor-2 (eIF-2α) at Ser 51—a conserved event activated in response to various cell stresses including hypoxia [28] at 20 h, suggesting that NK cells were stressed (Fig. 1c). To examine site-specific C>U editing in RNAs of NK cells, we selected several candidate genes including *TM7SF3* that we have previously shown high-level RNA editing on overexpressing A3G in 293T cells [17]. *TM7SF3* did not show any RNA editing in freshly isolated (T0/baseline) NK cells (Fig. 1d). However, we found evidence for the induction of RNA editing in *TM7SF3* due to cellular crowding with/without hypoxia (higher in hypoxia) (Fig. 1d), which did not further increase with IFN-γ treatment (Additional file 1: Figure S2a). Since A3G is also expressed in CD8+ T cells and to a lesser extent in CD4+ T cells (Fig. 1a, b), we cultured PBMCs as mentioned above and isolated NK, CD8+, and CD4+ cell subsets from the same donors. Site-specific RNA editing (> 5%) was observed in NK cells and to a lesser extent in CD8+ T cells, but not in CD4+ T cells (Fig. 1e), in parallel with the relative expression levels of A3G in these cell types. Since editing in NK and CD8+ T cells occurs in RNAs of genes that have been previously shown to be edited in the 293T/A3G overexpression system (*TM7SF3*, *RPL10A*, *RFX7*), our results suggest that A3G induces RNA editing in cytotoxic lymphocytes, particularly in NK cells.

### RNA-seq analysis of NK cells

To determine the transcriptome-wide targets of C>U RNA editing and their respective editing level in NK cells, we performed RNA-seq analysis. PBMCs (*n* = 3 donors) were cultured at a high density with/without hypoxia (1% O_2_), and site-specific editing of *TM7SF3* RNA was first confirmed, which showed a higher level of editing in hypoxia relative to normoxia (Fig. 1d). The three normoxic and three hypoxic NK cells’ RNA samples were then sequenced by following the TruSeq RNA Exome protocol (see the “Methods” section). To evaluate the quality of RNA editing detection, we initially compared all possible DNA-RNA nucleotide mismatches overrepresented in normoxia or hypoxia (FDR < 0.05; Additional file 1: Figure S2b). Hypoxic samples have more mismatches than normoxic samples for potential C>U (225 vs 93 C>T + G>A mismatches) and A>I (354 vs 126 A>G + T>C mismatches) RNA editing events as well as for all other mismatches (567 vs 394), indicating that DNA-RNA mismatches increase in hypoxia. This may be explained in part by the differences in RNA quality, which was lower in the hypoxic samples (see the “Methods” section). However, hypoxia increased putative C>U and A>I RNA editing events statistically significantly more than the other non-canonical mismatches (chi-squared, df = 1, *p* = 0.000183 and *p* = 0, respectively). This finding suggests that cellular crowding and hypoxia induces canonical C>U and A>I RNA editing events in NK cells.

To directly examine our hypothesis that hypoxia induces A3G-mediated RNA editing events in NK cells, we performed three successive filtering steps in the sequence data. The first filter (FDR < 0.05) identified all canonical C>U and A>I RNA editing events that were present at least 5% frequency in any sample and overrepresented in the hypoxia or normoxia group (Additional file 2: Table S1). The second filter (− 1 T or C) removed all C>U events that were not preceded by a T or C (Additional file 3; Table S2). The third filter (stem-loop) manually retained only the C>U sites that are located in a putative RNA stem-loop structure in exons or UTRs [27] (Additional file 4: Table S3; see the “Methods” section for details). The second and third filters aimed to eliminate C>U events that are less likely to be catalyzed by APOBEC3s but may represent rare genomic variants or false-positive results.

**Fig. 2:**
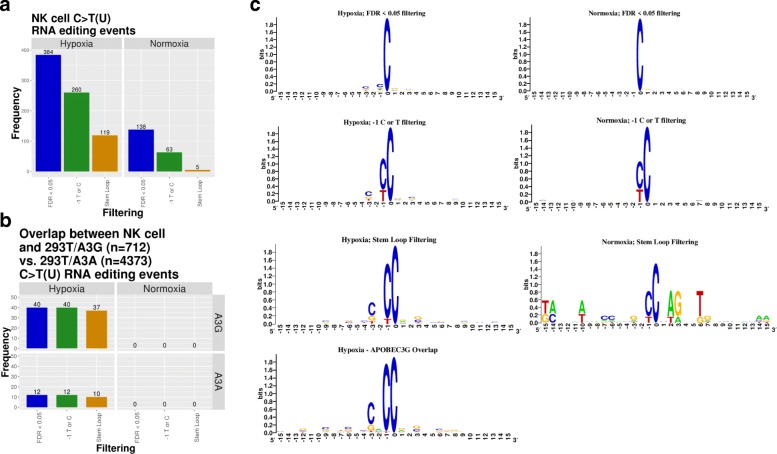
Summary of C>U RNA editing events in NK cells subjected to cellular crowding and normoxia/hypoxia. **a** Number of C>U RNA editing events higher in hypoxia vs higher normoxia after three successive filtering steps is shown. **b** Overlap of C>U RNA editing events between NK cell and 293T/A3G vs 293T/A3A overexpression systems is shown. **c** WebLogos show sequence conservation around the edited C (at position 0) among the edited RNAs, following each filtering step in hypoxic and normoxic, and among the 40 RNAs that are shared with 293T/A3G overexpression system. The height of nucleotides within the stack indicates the relative frequency of nucleic acid at that position

We then evaluated C>U RNA editing events overrepresented in normoxia or hypoxia (Fig. 2a). Hypoxia-induced C>U events outnumbered the normoxia-induced ones (384 vs 138), and they were more likely to have − 1 T or C (chi-squared, df = 1, *p* = 0.000005) and be located in putative stem-loop structure (Fisher’s exact test, *p* = 0). Furthermore, hypoxia-induced C>U events were more likely to overlap with sites previously identified in 293T/A3G (Additional file 5: Table S4) than those in the 293T/A3A overexpression system (Additional file 6: Table S5; Fig. 2b; Fisher’s exact test, *p* = 0). Four of 10 hypoxia-induced C>U editing events that were shared between hypoxic NK cells (after stem-loop filter) and the 293T/A3A system (Fig. 2b) were also present in the 293T/A3G system (Additional file 6: Table S5). These results strongly suggest that the contribution of A3A to hypoxia-induced RNA editing events in NK cells is minimal or absent. Stem-loop filtering step retains 37 of 40 sites overlapping with 293T/A3G while reducing the total editing events from 260 to 119 (2.18-fold) (chi-squared, df = 1, *p* = 0.00042), suggesting that stem-loop filtering enriches for true editing events by APOBEC3 enzymes. No overlap is seen between sites induced by normoxia in NK cells and the 293T/A3G or 293T/A3A overexpression systems. Upon successive filters, sequence motifs around the edited C (e.g., see preferred Cs at − 1 and − 3 positions in Fig. 2c) become increasingly similar to those seen in 293T/A3G overexpression system in hypoxia-induced, but not in normoxia-induced events (Fig. 2c and [17]). These results collectively support that APOBEC3-mediated RNA editing is induced by cellular crowding and hypoxia in NK cells and that A3G is likely the major contributor to these C>U editing events. Interestingly, 82 edited sites in hypoxic NK cells did not overlap with those in the 293T/A3G system. Cell type-specific factors and/or different mechanism of induction of RNA editing (exogenous overexpression versus crowding/hypoxia in primary cells) may play a role in targeting of distinct sites. We also find that 256 A>G(I) events are higher in hypoxia, of which 37 are present in the RADAR database [29] (Additional file 7; Table S6). In contrast, only 1 of 96 A>G(I) events higher in normoxia is present in RADAR database (Fisher’s exact test *p* < 0.00007; Additional file 1: Figure S3), suggesting that cellular crowding and hypoxia also induces bona fide A>I RNA editing events in NK cells.

**Fig. 3:**
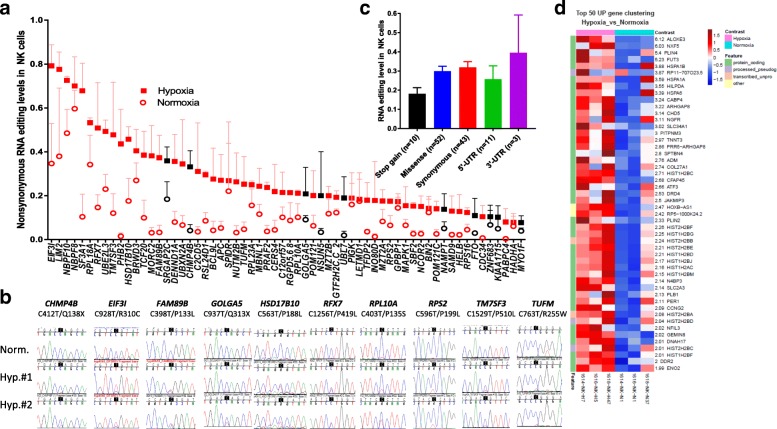
Induction of C>U RNA editing and gene expression in NK cells upon cellular crowding and hypoxia/normoxia. **a** C>U RNA editing in NK cells resulting in non-synonymous changes (*n* = 62) based in the order of highest to lowest editing level in hypoxia (40 h). Black symbols indicate genes that acquire nonsense RNA editing (*n* = 10). **b** Sanger sequence chromatogram traces of amplified cDNA fragments comparing site-specific C>U editing in mRNAs of ten genes under normoxia and hypoxia. **c** Graph representing the C>U RNA editing levels of based on the location and predicted mutational impact on protein. **d** Heat map representing the most upregulated genes (*n* = 50) in NK cells subjected to cellular crowding and hypoxia (cell stress)

RNA-seq analysis (after manual filters) revealed 119 site-specific C>U editing events in coding gene exons and UTRs which were edited at a higher level in hypoxia as compared to normoxia, although editing also occurred in normoxia at variable levels due to cellular crowding in NK cells (Fig. 3a, Additional file 4: Table S3). The largest group of editing events is comprised of non-synonymous changes, including 52 missense and 10 stop-gain changes (Fig. 3a). Synonymous C>U editing events occurred in RNAs of 43 genes (Additional file 1: Figure S2c). We verified RNA editing by Sanger sequencing of cDNAs in 10 of 10 non-synonymously edited genes, which include *CHMP4B*, *EIF3I*, *FAM89B*, *GOLGA5*, *HSD17B10*, *RFX7*, *RPL10A*, *RPS2*, *TM7SF3*, and *TUFM* (Fig. 3b). The highest level of non-synonymous RNA editing (~ 80%) occurred in *EIF3I*, which alters a highly conserved arginine to cysteine (c.C928T; R310C) (Fig. 3a, b). The average editing levels were lower for stop-gain changes than for missense, synonymous or UTR changes, suggesting functional constraints on editing events that introduce stop-gain changes (Fig. 3c). The levels of RNA editing and RNA expression in hypoxia show no correlation (*r* = 0.1068, *p* = 0.2476, *n* = 119 genes, Additional file 1: Figure S4), suggesting that expression level of the RNA edited genes is not a major factor on RNA editing levels. Gene expression analysis of APOBEC genes revealed A3G as the most expressed gene and no evidence of induction by hypoxia (Additional file 1: Figure S5).

We determined the functional clustering of genes that undergo non-synonymous changes (*n* = 62) due to RNA editing in NK cells using DAVID Bioinformatics Resources [30]. The highest enrichment was in genes for “translation initiation,” “translation,” and “ribosome” (Additional file 1: Figure S6) due to missense changes in RNAs of eight genes (Table 1), including the highest non-synonymously edited *EIF3I* (Fig. 3a). RNA editing targeted highly conserved amino acids in seven of eight genes as predicted by at least two of the three softwares (Table 1 and see Additional file 8: Table S7 for conservation analysis of all non-synonymous RNA editing sites). RNA editing also altered a conserved C (phyloP100 score = 1.7811) at − 4 nucleotide position in the 5′-UTR of another gene encoding the ribosomal protein, *RPLP0* (Additional file 4: Table S3). Since the regulation of translation plays a central role during cell stress [31, 32], these results suggest that RNA editing coordinately alters multiple ribosomal and other translational proteins and may have an impact on the quality or quantity of protein translation under hypoxic stress.

**Table 1:** Evolutionary conservation of C>U recoding RNA editing sites in translational and ribosomal genes

Mutation	Gene	AAChange	PolyPhen	SIFT	PhiloP
1:32231146-CT	*EIF3I*	NM_003757:exon11:c.C928T:p.R310C	Possibly damaging (0.901)	Deleterious (0)	2.64453
15:55196824-GA	*RSL24D1*	NM_016304:exon1:c.C67T:p.R23C	Benign (0.011)	Deleterious (0.02)	3.43396
6:35470271-CT	*RPL10A*	NM_007104:exon5:c.C403T:p.P135S	Possibly damaging (0.866)	Deleterious (0.01)	7.64955
19:17863201-CT	*RPL18A*	NM_000980:exon5:c.C469T:p.R157W	Benign (0.12)	Deleterious (0.03)	3.41186
17:28720820-CT	*RPL23A*	NM_000984:exon2:c.C139T:p.R47W	Benign (0.013)	Tolerated (0.28)	1.92739
19:39433341-GA	*RPS16*	NM_001020:exon5:c.C373T:p.R125C	Possibly damaging (0.901)	Deleterious (0.03)	7.59689
16:1962610-GA	*RPS2*	NM_002952:exon6:c.C596T:p.P199L	Possibly damaging (0.905)	Deleterious (0.03)	9.862
1:39561754-GA	*PABPC4*	NM_001135653:exon15:c.C1927T:p.H643Y	Benign (0.063)	Deleterious (0.02)	10.003

We also examined the changes in gene expression that occur during the induction of RNA editing in NK cells due to cellular crowding and hypoxia. We found upregulation of 82 genes and downregulation of 237 genes (fold change > 2 and padj < 0.05; Additional file 1: Figure S7 and Additional file 9: Table S8). Multiple genes of the heat shock protein HSP70 family (*HSPA1B*, *HSPA1A*, *HSPA6*) [33] and *ATF3*, which encodes a transcription factor integral to the ER stress response [34] are among the most upregulated (Fig. 3d). Thus, cellular crowding and hypoxia trigger coordinated transcriptome remodeling in NK cells, which includes transcriptional induction of stress genes as well as recoding C>U RNA editing of translational and ribosomal genes.

### Confirmation of APOBEC3G-mediated RNA editing in lymphoma cell lines

To confirm A3G-mediated RNA editing and to examine the functional consequence of this editing in a cell line, we searched for cell lines that express A3G. The highest expression of A3G was observed in leukemia and lymphoma cell lines at the CCLE database [35] (see Additional file 1; Figure S8). We studied HuT78, a CD4+ cutaneous T cell lymphoma cell line, which was previously used to identify A3G as a restriction factor for vif-deficient HIV-1 [16], and JVM2, a B cell mantle cell lymphoma cell line carrying t(11;14)(q13; q32) translocation [36]. Examination of expression data in two datasets deposited in the GEO database [37] suggested that A3G was the highest expressed APOBEC3 gene in both cell lines (see Additional file 1; Figure S9). This finding is consistent with an earlier study which showed that A3G was the highest expressed APOBEC3 gene in H9 cell line, a derivative of Hut78 [26]. As expected, both Hut78 and JVM2 lymphoma cell lines showed evidence of A3G-mediated RNA editing in response to high cell density and hypoxia (Additional file 1: Figure S9).

**Fig. 4:**
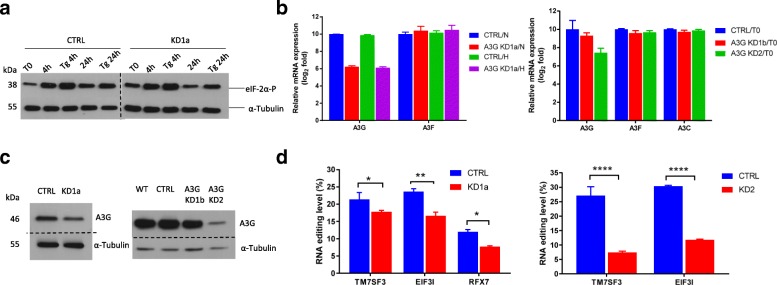
Knockdown of A3G reduces C>U RNA editing induced by cellular crowding in HuT78 cell line. **a** Immunoblot showing the protein levels of eIF-2α phosphorylated at Ser 51 in whole cell lysates of scramble CTRL and KD1a cells at various time points. Thapsigargin (Tg)-treated HuT78 cells is a positive control, and α-tubulin is used as a loading control. **b** *A3G*, *A3F*, and *A3C* gene expression in control and KD HuT78 cells by RT-qPCR. Gene expression measurements are normalized to that of β2-microglobulin. Expression of each gene is arbitrarily set to ten in control (CTRL) cell line to highlight specific targeting of A3G. **c** Immunoblot for A3G protein expression in whole cells lysates of WT (original wild-type cell line), scramble CTRL, and A3G KD HuT78 cells shows a reduction in A3G protein levels in KD1a and KD2. α-Tubulin is used as a loading control. **d** Graph representing the percentage site-specific C>U RNA editing level for *TM7SF3*, *EIF3I*, and *RFX7* of scramble CTRL and KD HuT78 cells in normoxia with cellular crowding (*n* = 3, mean and SEM). N, normoxia with cellular crowding; H, hypoxia with cellular crowding; T0, normoxia without cellular crowding. See the “Methods” section for statistical analysis

As compared to primary CD4+ T cells, A3G is highly expressed in the HuT78 lymphoma cell line (Fig. 1b). To further validate the RNA editing function of A3G, we knocked down A3G in these cells using A3G-specific shRNA lentiviral constructs (initially with KD1a and subsequently with KD1b and KD2 constructs as described in the “Methods” section) and a scramble negative control shRNA (referred to as CTRL HuT78). To examine the RNA editing function of A3G, the CTRL and the A3G knockdown HuT78 cell lines were cultured at a high density of 1 × 10^6^ cells in 100 μl per well in a 96-well plate for 24 h in normoxia or hypoxia (1% O_2_). High-density culture and/or hypoxia treatment induced cell stress as there was an increased accumulation of phosphorylated eIF-2α [28], 4 h post-culture (Fig. 4a). Under these conditions, we measured the expression of *A3G* in these cell lines by qPCR (Fig. 4b). The KD HuT78 cells showed reduced expression of *A3G* as compared with CTRL HuT78 (Fig. 4b). We did not observe any significant variation in *A3G* levels with or without hypoxia treatment in the CTRL and KD HuT78 cells. *A3F* and *A3C* which is also expressed in HuT78 cells at lower levels (Additional file 1: Figure S9) did not show any significant variation in expression level between the CTRL and KD HuT78 cell lines, indicating that the knockdown for A3G is specific (Fig. 4b). We further confirmed the knockdown of *A3G* by analyzing its protein expression by western blot (Fig. 4c) using specific antibodies to A3G. As compared to CTRL, KD2 and KD1a shRNA constructs caused severe and moderate reduction in A3G protein expression, respectively (Fig. 4c).

To determine the effect of A3G knockdown on RNA editing, we analyzed the editing level of three RNAs (*TM7SF3*, *EIF3I*, and *RFX7*) previously validated as editing targets in NK cells. When cultured at a high density (mentioned above), we found site-specific editing of *TM7SF3*, *EIF3I*, and *RFX7* RNAs in CTRL HuT78 cells and the level of editing was reduced in the A3G KD1a and KD2 HuT78 cells (Fig. 4d and Additional file 1: Figure S10), correlating with the expression of A3G in these cells. Notably, editing levels in KD2 HuT78 cells were close to 5% detection threshold, indicating that A3G is required for site-specific deamination of these transcripts.

Considering that (1) A3G has a CC nucleotide preference, (2) RNA editing targets in NK cells and in 293T/A3G overexpression system overlap significantly, (3) the same RNAs are site-specifically edited in NK and HuT78 cells-both highly expressing A3G, and (4) A3G KD HuT78 cells show decreased RNA editing; these results collectively indicate that A3G is an endogenous, inducible mRNA editing enzyme in NK, CD8+, and HuT78 (and JVM2) cells.

### A3G induces RNA editing by mitochondrial respiratory inhibition, independently of HIF-1α

**Fig. 5:**
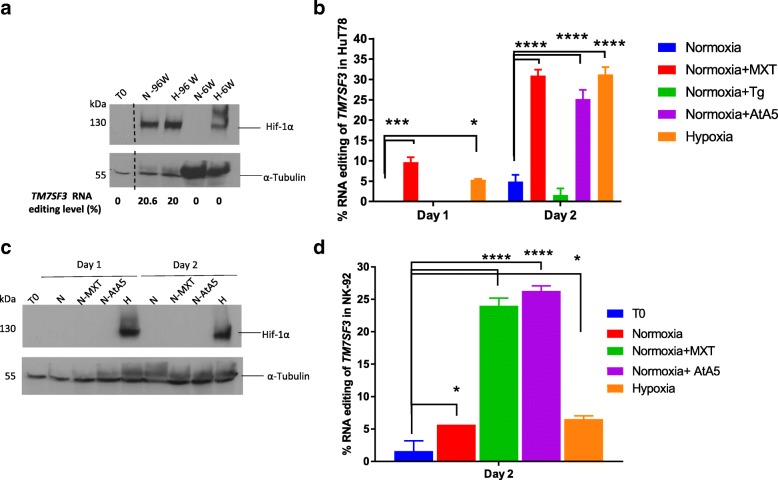
Induction of A3G-mediated C>U mRNA editing by the inhibition of mitochondrial respiration. **a** Immunoblot showing the protein level of HIF-1α in whole cell lysates of HuT78 when subjected to normoxia (N) and hypoxia (H) in 96-well (W) and 6-W plates for 24 h. All lanes are part of the same gel. The dashed line represents the cropped region. The percentage of C>U RNA editing levels in *TM7SF3* under these conditions is displayed below. **b** The percentage of C>U RNA editing in *TM7SF3* when HuT78 cells are treated with myxothiazol (MXT), thapsigargin (Tg), Atpenin (AtA5), and hypoxia (H) for 24 h (day 1) or 42 h (day 2) (*n* = 3). **c** Immunoblot showing the protein level of HIF-1α in whole cell lysates of HuT78 when subjected to normoxia (N) with or without the mitochondrial inhibitors (MXT and AtA5) and hypoxia (H) for 1 or 2 days. **d** The percentage of C>U RNA editing in *TM7SF3* when NK-92 cells are treated with myxothiazol (MXT), Atpenin (AtA5), and hypoxia (H) for 42 h (*n* = 3). See the “Methods” section for statistical analysis

To determine whether high density of HuT78 cells, which induces RNA editing by A3G, causes hypoxia, we cultured 1 × 10^6^ HuT78 cells in 100 μl per well in 96-well plates (high density) and the same number of cells in 1 ml culture in 6-well plates (low density), each under normoxia and hypoxia. We analyzed the stabilization of the hypoxia-inducible factor-1α (HIF-1α) protein, which is well known to be stabilized in hypoxic cells to promote the synthesis of mRNAs involved in cellular homeostasis [38], and measured the RNA editing levels of *TM7SF3*. As expected, HIF-1α was not stabilized at T0− when the cells were at a non-stressed state or under low-density normoxic cell culture (6-well) after 24 h (Fig. 5a). However, we found the stabilization of HIF-1α in cells cultured at a high density in 96-well plates both in normoxia and hypoxia and in cells cultured at a low density in 6-well plates in hypoxia, suggesting that the high density 96-well normoxic culture had turned hypoxic (Fig. 5a). Under these conditions, RNA editing of *TM7SF3* was observed in cells cultured at a high cell density in both normoxia (20.6%) and hypoxia (20%) (Fig. 5a; Additional file 1: Figure S9). Although HIF-1α stabilization was observed in low cell density (6-well) hypoxic cultures, no RNA editing was observed under these conditions (Fig. 5a). These results confirm that as in NK cells, RNA editing is induced by high cell density and hypoxia in HuT78 cells. Moreover, the stabilization of HIF-1α is not sufficient for the induction of RNA editing.

Previously, we have shown that A3A-mediated RNA editing is induced by high cell density and hypoxia in hundreds of mRNAs in monocytes [15]. Furthermore, normoxic inhibition of the mitochondrial complex II by atpenin A5 (AtA5) and of the complex III by myxothiazol (MXT) mimics hypoxia and induces RNA editing as well as hypoxic gene expression in monocytes [39]. Since A3G-mediated RNA editing in NK and HuT78 cells is also induced by hypoxia, we tested the effect of these mitochondrial inhibitors on RNA editing in HuT78 cells cultured in normoxia. Additionally, to test whether endoplasmic reticulum (ER) stress can also induce RNA editing, we treated the cells with thapsigargin (Tg). Tg induces ER stress by raising intracellular calcium levels and lowers the ER calcium levels by specifically inhibiting the endoplasmic reticulum Ca^++^ ATPase [40, 41], resulting in the accumulation of unfolded proteins and an increased accumulation eIF-2α phosphorylated at Ser 51 (Figs. 1c and 4a). To test the effect of hypoxic stress alone on HuT78 cells, we reduced the cell density to avoid cellular crowding and cultured the cells at an intermediate density of 0.5 × 10^6^ cells per 500 μl per well in 24-well plates with or without the chemical inhibitors in normoxia and hypoxia alone for 1 or 2 days. Under these conditions, we determined the RNA editing level and the stabilization of HIF-1α in these cells. We observed that RNA editing is mildly induced in cells treated with MXT and by hypoxia alone on day 1, at approximately 10% and 5% levels, respectively (Fig. 5b). RNA editing levels increased to approximately 30% in cells treated with MXT, AtA5, or hypoxia alone on day 2. Treatment of cells with Tg did not induce RNA editing (Fig. 5b). Furthermore, HIF-1α was stabilized only when the cells were subjected to hypoxia but not in normoxia in the presence or absence of the mitochondrial inhibitors (Fig. 5c). These results suggest that RNA editing induced by hypoxic stress at a high cell density is triggered by mitochondrial respiratory inhibition and occurs independently of the stabilization of HIF-1α as well as the ER stress response.

NK-92 lymphoma cell line is derived from NK cell lymphoma and is used in cancer immunotherapy [42]. Given its similar characteristics to primary NK cells and the convenience of culturing NK-92 cells as compared with primary NK cells, we tested the induction of RNA editing in NK-92 cells. We treated NK-92 cells with normoxia with or without the mitochondrial inhibitors (AtA5 or MXT) or hypoxia alone at an intermediate density in 24-well plates for 2 days. Interestingly, RNA editing was induced by the inhibition of mitochondrial respiration (~ 25%) but only slightly by hypoxia treatment (Fig. 5d) in NK-92 cells. The reason behind the difference in hypoxia-induced RNA editing level of HuT78 and NK-92 cells may be due to the metabolic differences between the two cell lines. However, the induction of A3G-mediated RNA editing due to mitochondrial respiratory stress in NK-92 cells provides a model system and an opportunity for further functional studies related to NK cells.

### APOBEC3G promotes Warburg-like metabolic remodeling without inhibiting cell proliferation under stress

We have previously identified *SDHB* and *SDHA* mitochondrial complex II subunits as targets of A3A-mediated RNA editing in hypoxic monocytes [15]. In the current study, we find that A3G non-synonymously edits several mitochondrial genes’ RNAs including *TUFM*, *HADHA*, *HSD17B10*, and *PHB2* in hypoxic NK cells (Fig. 3a). Thus, we hypothesized that hypoxic stress-induced RNA editing by A3G alters mitochondrial function.

**Fig. 6:**
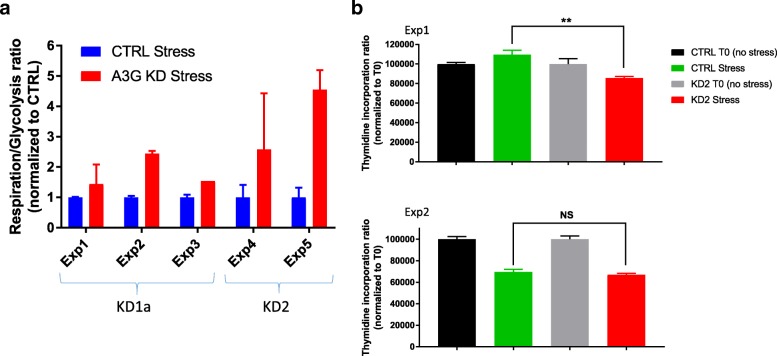
A3G suppresses mitochondrial basal respiration relative to glycolysis without inhibiting proliferation in crowded HuT78 cells. **a** Respiration/glycolysis (R/G) ratios, first normalized to T0 unstressed controls, then to CTRL, which is set to 1, in five different Seahorse experiments show increased R/G ratio by A3G KD (*p* = 0.004 by *t* test, total *n* = 15 in five experiments). Exp1–3 are performed with KD1a; Exp4 and5 are performed with KD2. Seahorse assay is performed on unstressed (no editing) and stressed (after cellular crowding with 5–10 million/ml) in normoxia, which induces RNA editing. (*n* = 2–4 replicates per experiment; mean and SEM shown). **b** Cell proliferation changes upon induction of RNA editing by cellular crowding in CTRL, and KD2 HuT78 cells measured by thymidine incorporation assays. CTRL and KD T0 values are normalized to 10,000 CPM, and relative changes caused by crowding are shown. (*p*(exp1) = 0.008, *p*(exp2) = 0.41 by unpaired *t* test. Fisher’s combined probability test *p* value < 0.05, χ^2^ = 11.44, df = 4)

To test the role of A3G on bioenergetics in response to high cell density and hypoxic stress, we measured the metabolic profile of CTRL and KD HuT78 cells using the Seahorse Platform. We performed the mitochondrial and the glycolytic stress tests to measure the oxygen consumption rate, representative of basal respiration, and the extracellular acidification rate, representative of glycolysis in cells cultured at a high density under normoxia (Fig. 6a). We have presented metabolic alterations as a respiration-to-glycolysis ratio (R/G) both in unstressed (T0) and stressed cells (crowding) after normalizing for unstressed T0 cells first, then to the ratio observed in CTRL cells in each experiment (raw data for each experiment are presented in Additional file 1: Figure S11.). We have observed a higher R/G ratio by both A3G KD1a and KD2 constructs under the stress conditions that induce RNA editing, suggesting that cellular RNA editing by A3G reduces the R/G ratio. An independent evaluation by WST-8-based dye assay further supported higher dehydrogenase activity, which is primarily contributed by mitochondria, in A3G KD1a cells compared to CTRL (see Additional file 1: Figure S11). Higher R/G ratios observed in both KD1a and KD2 HuT78 cells indicate that cellular RNA editing by A3G promotes Warburg-like metabolic remodeling by suppressing mitochondrial respiration relative to glycolysis even in the presence of residual oxygen.

To examine the role of A3G on cellular proliferation under stress, we measured the proliferation of the CTRL and KD2 HuT78 cells after RNA editing is induced by cellular crowding in normoxia. Cell proliferation is measured directly by thymidine incorporation assay. We find that RNA editing by A3G did not reduce cell proliferation compared to A3G KD2 since slightly higher levels of proliferative activity are seen in CTRL cells relative to A3G KD2 in which RNA editing is significantly impaired (Fig. 6b).

## Discussion

In this study, we find that A3G edits scores of RNAs in NK cells and to a lesser extent in CD8+ T lymphocytes as well as lymphoma cell lines, when cultured at a high density and hypoxia. A3G-mediated site-specific RNA editing is triggered by the inhibition of mitochondrial respiration and targets the mRNAs of many ribosomal and translational genes resulting in non-synonymous changes. A3G reduces mitochondrial respiration relative to glycolysis without inhibiting cell proliferation under stress in transformed lymphoma cells (Fig. 6). These results identify A3G cytidine deaminase as the third endogenous C>U RNA editing enzyme in mammals and together with A3A in myeloid cells, defines a new functional category of RNA editing enzymes that are activated by certain stress in immune cells. In addition, our findings uncover a previously unrecognized gene regulation mechanism in primary NK cells and lymphoma cell lines that are induced by hypoxic stress.

There are two major differences in A3-mediated RNA editing and ADAR- and APOBEC1-mediated editing. First, A3-mediated RNA editing is induced upon hypoxic stress (A3A and A3G) or by IFNs (A3A), while it is essentially absent or rare in baseline unstressed immune cells [15] (Fig. 1d). In contrast, ADAR- and APOBEC1-mediated RNA editing events occur in baseline unstimulated cells [43–45]. Second, A3-mediated RNA editing events occur in exonic coding regions of genes as commonly as in UTRs ([15] and Fig. 3c), whereas ADAR- and APOBEC1-mediated RNA editing events preferentially occur in UTRs, where they are at least an order of magnitude more frequent relative to coding exons [43–45]. Together, these findings suggest that A3-mediated RNA editing plays a role in response to certain cell stress by altering protein function.

A recurrent theme in many types of cell stress responses, including ER and mitochondrial unfolded protein stress response generally caused by heat shock, nutrient deprivation, hypoxia, or DNA damage, is the regulation of gene expression. This is achieved by the general suppression or reprogramming of translation to promote recovery from stress or cell death [32, 46]. We observe the highest level of RNA editing resulting in a non-synonymous change in *EIF3I*. *EIF3I* encodes a subunit of EIF3, the most complex translation initiation factor comprised of 13 subunits in mammals, which is involved in all molecular aspects of translation initiation. The EIF3 complex has been implicated in the translation of mRNAs important for cell growth [47] and mitochondrial respiration [48], and its subunits are overexpressed in multiple cancers [49]. Interestingly, EIF3I was previously shown to have decreased protein synthesis in cold-stressed mammalian cells, implying its important role in stress response and recovery [50]. Consistent with these reports, we find that the knockdown of A3G in HuT78 lymphoma cells reduces the predicted deleterious RNA editing of *EIF3I* in association with increased mitochondrial respiration relative to glycolysis during hypoxic stress. Thus, our findings suggest that A3G promotes hypoxic stress responses via RNA editing of *EIF3I*, ribosomal/translational genes, and possibly other stress-related genes.

Cancer cells switch to aerobic glycolysis even in the presence of functional mitochondria, and this phenomenon is termed the “Warburg effect.” However, the function of the Warburg effect in tumor growth, proliferation, and support of cellular biosynthetic programs is still inconclusive [51]. In response to acute hypoxia, A3G-mediated RNA editing may promote Warburg effect by preferring glycolysis over mitochondrial respiration and decreased translation. Warburg-like metabolic remodeling is thought to promote cellular proliferation in bacteria and cancer cells [52]. RNA editing by A3G may play a role in supporting proliferation under hypoxic stress by promoting Warburg-like remodeling in lymphoma cells, although the mechanisms linking edited genes to proliferation require further investigations. We find slightly lower levels of cell proliferation upon A3G KD2 after crowding stress in vitro (Fig. 6b), although the impact of A3G on lymphoma cell proliferation requires further studies, especially in in vivo conditions.

Interestingly, even though normal B cells and plasma cells show low expression of A3G (Fig. 1a), we find the highest expression levels in neoplastic B and plasma cell lines derived from acute lymphoblastic leukemia, B cell lymphoma, Burkitt lymphoma, and multiple myeloma [35]. Increased expression of A3G in many B cell leukemia/lymphoma cell lines, and NK/T cell lymphoma [53] supports the notion that A3G may play an oncogenic role by enhancing survival and/or proliferation under oxygen-limiting conditions caused by rapid and uncontrolled cell divisions. It is known that NK cell function is impaired in the tumor microenvironment or chronic infections due to multiple factors, including hypoxia [54]. Since A3G profoundly alters the coding transcriptome of NK cells under hypoxic stress, it may play an important role in regulating NK cell anti-tumor activity in the tumor microenvironment.

**Fig. 7:**
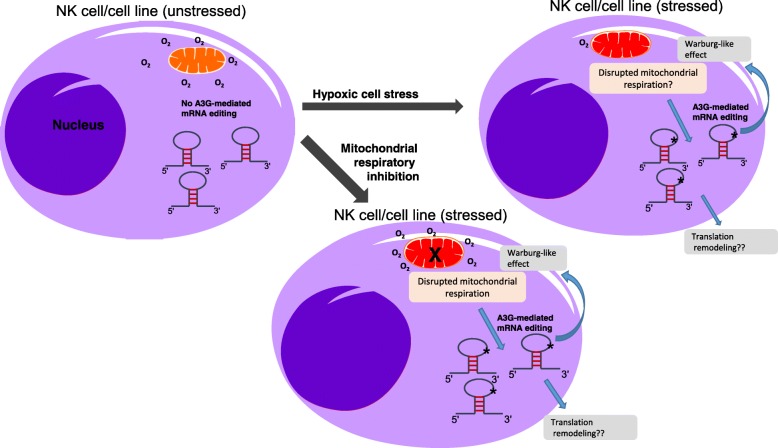
Simplified diagram summarizing the induction and relevance of A3G-mediated site-specific C>U cellular mRNA editing in NK cells and lymphoma cell lines. NK/lymphoma cell is shown under normal physiological conditions when the cells are unstressed (left) or when the cells are stressed by hypoxia (top right) or due to the inhibition of mitochondrial respiration (bottom right). Under normal physiological conditions (baseline), mRNAs (stem-loop) in NK cells do not undergo C>U RNA editing. Under hypoxic stress or upon mitochondrial respiratory inhibition, an unknown signal originating in the mitochondria triggers site-specific A3G-mediated C>U editing in multiple mRNA substrates bearing a stem-loop structure. The cellular mRNA editing induced by mitochondrial hypoxic stress may result in translational reprogramming of NK cells, Warburg-like metabolic remodeling by preferring glycolysis over mitochondrial respiration and support cellular proliferation in order to promote adaption during NK/lymphoma cell stress

We also find that RNA editing by A3G can be induced by normoxic inhibition of mitochondrial respiration and occurs independently of HIF-1α stabilization (Fig. 5), in a manner similar to the regulation of A3A-mediated RNA editing in monocytes [39]. Earlier studies have shown that the inhibition of mitochondrial respiration antagonizes the stabilization of HIF-1α in hypoxia (reviewed in [55]). Despite the lack of HIF-1α stabilization, however, we find that mitochondrial respiratory inhibition mimics hypoxic stress and induces RNA editing by A3G. Thus, A3G-mediated RNA editing joins a growing number of hypoxia-induced responses that can be mimicked by the inhibition of mitochondrial respiration. These include carotid body paragangliomas caused by mitochondrial complex II mutations [56], expression of hypoxia-related genes and A3A-meditated RNA editing responses in monocytes [39], stimulation of the cardiorespiratory system by carotid body chemoreceptors [57], hypoxic pulmonary vasoconstriction mediated by pulmonary arterial smooth muscle cells [58], and hypoxia-induced changes in astrocytes [59], the most abundant glial cells in the brain. We hypothesize that hypoxia triggers A3G-mediated RNA editing by activating a pathway triggered by mitochondrial respiratory inhibition as a result of severe oxygen deprivation or respiratory inhibitors in normoxia (Fig. 7). Since A3G transcript levels do not increase when RNA editing is induced upon cellular crowding and hypoxia (Additional file 1: Figure S5), the mechanism may involve post-translational changes activating A3G leading to altered intracellular trafficking, protein-protein interactions, or another dynamic pathway. Details of this mitochondrial hypoxic signaling pathway that activates A3-mediated RNA editing are subject of future studies. Furthermore, we cannot rule out the possibility that cellular crowding may also trigger additional pathways involving reduced cytokines, growth factors, amino acids, or other nutrients that collaborate with hypoxia to induce RNA editing.

Finally, the unexpected discovery of RNA editing functions for A3A and A3G requires reconsideration of the physiological functions of the A3 enzymes solely as anti-viral factors. For example, A3G evolved with positive selection signature for millions of years in the primate lineage before humans were infected by HIV-1 [60]. Also, A3G orthologs that have the signature of positive evolutionary selection are present in primates that are not infected by SIVs [61]. Although suppression of endogenous retroviruses was speculated as an in vivo function of A3 enzymes, mouse A3 knockout is viable without any evidence of catastrophic retroviral infection/reactivation [62]. Furthermore, the anti-HIV model of the double-domain A3G does not adequately explain why the zinc-coordinating residues in the N-terminal domain are conserved, since ssDNA deamination of HIV-1 minus strand by A3G in target cells does not require catalytic activity of the N-terminal domain [13, 14]. In contrast, RNA editing requires the conserved zinc-coordinating residues in both its N- and C-terminal domains [17]. Thus, cellular RNA editing provides a plausible explanation for A3G’s long-term evolutionary history, the presence of two conserved zinc-coordinating catalytic domains and the high expression patterns in NK cells and lymphoma cell lines. In conclusion, our findings suggest that the primary function of A3G in vivo may be cellular RNA editing to facilitate adaptation to mitochondrial hypoxic stress in innate lymphocytes. Further studies are required to examine the RNA editing function of the other APOBEC3 enzymes, as well as their significance in immunity.

## Conclusion

This study shows the endogenous inducible site-specific RNA editing activity of the A3G cytidine deaminase, the most studied member of the APOBEC3 family, and suggests its physiological function in human NK and transformed lymphoid cells. Widespread RNA editing by A3G can facilitate cellular adaptation to hypoxic cell stress triggered by mitochondrial respiratory inhibition in primary cytotoxic lymphocytes and in lymphoma cell lines. A3G is the third endogenous C>U RNA-editing enzyme to be identified in mammals. In addition, our study uncovers a novel epitranscriptomic gene regulation mechanism in cytotoxic lymphocytes, specifically NK cells. APOBEC3 cytidine deaminases may define a new class of RNA editing enzymes that are activated in response to certain cell stress factors.

## Methods

### RNA sequencing

RNAs (DNA-free) were extracted from NK cells of 3 donors subjected to normoxia and hypoxia treatments (6 samples total) using the Total RNA clean-up and concentration kit (Norgen Biotek) as per the manufacturer’s instructions. RNA libraries were prepared using the Illumina TruSeq RNA Exome protocol and kit reagents. RNA input for intact total RNA was 10 ng. RNA QC analysis by electrophoresis (2100 Expert, B.02.08.SI648, Agilent Technologies, Inc.) showed RIN numbers of 9.6, 7.8, and 6.4 for normoxic and 2.8, 9.4, and 2 for hypoxic samples. These RIN numbers showed evidence of RNA degradation. Therefore, for degraded RNA samples, input amount was determined by calculating the percentage of RNA fragments > 200 nt (DV200) by running the samples on an RNA ScreenTape (Agilent Technologies) and performing region analysis using the Tapestation Analysis Software. Based on the DV200 calculation of 52–85%, 40 ng was the input amount and was considered suitable for this protocol. Fragmentation of the RNA was performed on intact samples. First and second strand syntheses were performed to generate double-stranded cDNA. The 3′-ends were adenylated and Illumina adapters were ligated using T-A ligation. PCR was performed to generate enough material for hybridization and capture. PCR products were validated for the correct sizing using D1000 Screentape (Agilent Technologies). Two hundred nanogram of each product was pooled together in 4-plex reactions for hybridization and capture. Two sequential rounds of hybridization and capture were performed using the desired Capture Oligo pool. The second round of PCR was done to generate sufficient libraries for sequencing. Final libraries were validated for correct size distribution on a D1000 Screentape, quantified using KAPA Biosystems qPCR kit, and the 4-plex capture pools were pooled together in an equimolar fashion, following experimental design criteria.

Each pool was denatured and diluted to 2.4 pM with 1% PhiX control library added. Each pool was denatured and diluted to 16 pM for On-Board Cluster Generation and sequencing on a HiSeq2500 sequencer using 100-cycle paired-end cluster kit and rapid mode SBS reagents following the manufacturer’s recommended protocol (Illumina Inc.) and 100 million paired reads per sample were obtained. The sequence data from 6 samples (3 normoxic and 3 hypoxic) from 3 donors are deposited in GEO [63].

### RNA editing bioinformatics analysis

#### RNA editing events detection

Sequence reads passing quality filter from Illumina RTA were first checked using FastQC [64] and then mapped to GENCODE (https://www.gencodegenes.org/) annotation database (V25) and human reference genome (GRCh38.p7) using Tophat2 [65] with a lenient alignment strategy allowing at most two mismatches per read to accommodate potential editing events. The mapped bam files were further QCed using RSeqQC [66]. Then, all samples were run through the GATK best practices pipeline of SNV calling (https://gatkforums.broadinstitute.org/gatk/discussion/3892/the-gatk-best-practices-for-variant-calling-on-rnaseq-in-full-detail) using RNA-seq data to obtain a list of candidate variant sites. All known SNPs from dbSNP (V144) [67] were removed from further analyses.

#### Hypoxia/normoxia-induced editing event filtering

Pileups at candidate sites were generated using SAMtools for all samples, and the base counts for alternative and reference base were calculated. Potential candidates for RNA editing were first filtered using the following two criteria: (a) at least 5% editing level on any sample within the population and (b) only C>T/G>A and A>G/T>C events were selected. The editing base counts were modeled as binomial distribution, and the effect of hypoxia/normoxia on RNA editing at each site was tested with a generalized linear model (GLM) using paired samples. Multiple test adjustment was applied using the Benjamini-Hochberg procedure to control false discovery rate (FDR). Hypoxia- and normoxia-induced editing events were identified with log odds ratio greater than 0 and adjusted *p* value less than 0.05.

#### Results

A table specifying the editing site, type of editing event, editing level and number of reads on a reference, and alternative bases on each sample for each group was initially produced filtering events with OR > 1 and a FDR < 0.05 level.

#### Annotation

Hypoxia/normoxia-induced editing events passing filters were annotated using ANNOVAR [68] with RefSeq gene annotation database to identify gene features, protein changes, and potential impact. Also, 15 bp upstream and downstream flanks from the variant sites were displayed in separate columns.

#### Manual filters

The above analyses initially revealed 384 C>U editing sites which were then subjected to 2 filtering steps which retained only those sites (1) with − 1 position (relative to edited C) either a C or T (manual filter) and (2) within exons and UTRs, and a putative stem-loop structure where the edited C is at the 3′-end of a putative tri- or tetra loop which is flanked by a stem that was at least 2 bp long when base complementarity was perfect, or at least 4 bp long when complementarity was imperfect by 1 nucleotide mismatch or 1 nucleotide bulging. These filters reduced the number of edited sites to 119.

### RNA-seq differential expression analysis

Raw counts for each gene were generated using HTSeq [69] with intersection_strict mode. Differential gene expression was analyzed by DESeq2 [70]. Bioconductor package with paired sample design to identify hypoxia-induced gene expression changes.

### Conservation analysis of amino acids recoded by RNA editing in NK cells

The impact of non-synonymous RNA editing on protein function was examined by PolyPhen and SIFT programs from ENSEMBL VEP tool, which give a score and a verbal description of the impact [71]. In addition, the conservation score based on 100 vertebrates base-wise conservation was obtained from UCSC (phyloP100way) [72].

### Isolation and culture of cells

The HuT78, JVM2, and NK-92 cell lines were obtained from ATCC. HuT78 cells used in A3G KD2 experiments were purchased from Sigma-Aldrich. HuT78 cells were cultured in IMDM (ATCC) containing 20% fetal bovine serum (FBS) (Sigma-Aldrich), JVM2 cells were cultured in RPMI (ATCC) containing 10% FBS, and NK-92 cells were cultured in alpha minimum essential medium without ribonucleosides and deoxyribonucleosides (Life Technologies) but with 2 mM l-glutamine and 1.5 g/l sodium bicarbonate as well as 0.2 mM inositol, 0.1 mM 2-mercaptoethanol, 0.02 mM folic acid, 500 U/ml IL-2 (aldesleukin—a kind gift from Novartis), 12.5% horse serum (ATCC), and 12.5% FBS. Peripheral blood mononuclear cells (PBMCs) of anonymous platelet donors were isolated from peripheral blood in Trima Accel™ leukoreduction system chambers (Terumo BCT) in accordance with an institutional review board-approved protocol, as described earlier [15], in RPMI-1640 medium (Mediatech) with 10% FBS, 100 U/ml penicillin, and 100 μg/ml streptomycin (Mediatech). NK, CD4+, and CD8+ cells were isolated from human PBMCs (cultured at 5 × 10^7^ in 1.8 ml per well in 12-well plates) by immunomagnetic negative selection using the EasySep™ Human NK Cell Isolation Kit (Stemcell Technologies, catalog # 17955), EasySep™ Human CD4+ Cell Isolation Kit (Stemcell Technologies, catalog # 17952), and EasySep™ Human CD8+ Cell Isolation Kit (Stemcell Technologies, catalog # 17953), respectively, following the manufacturer’s instructions. Enrichment for NK cells was > 90% (Additional file 1: Figure S12) and that of CD4+ and CD8+ was > 99%, as verified by flow cytometry.

### Cell stress and inhibitor treatment

For cell crowding experiments, the HuT78 cells were cultured at a density of 0.5–1 × 10^6^ cells per 100 μl per well in 96-well plates for 22–24 h at 37 °C.

For hypoxia treatment, PBMCs were cultured at a density of 5 × 10^7^ in 1.8 ml per well in 12-well plates under 1% O_2_, 5% CO_2_, and 94% N_2_ in an Xvivo™ System (Biospherix) for 40 h. Following culture, NK, CD4+, and CD8+ cells were separated as mentioned above. In case of HuT78, the cells were cultured in the hypoxia chamber for 24 or 40 h at a density of 1 × 10^6^ cells per ml in 6-well plates.

For testing the mitochondrial inhibitors, HuT78 and NK-92 cells were cultured at 0.5 × 10^6^ cells per 0.5 ml in 24-well plates in normoxia with or without AtA5 and MXT or hypoxia alone for 2 days at 37 °C.

Human IFN-γ was obtained from PeproTech and used at a concentration of 50 ng/ml. AtA5 (Cayman chemical #11898) and MXT (Sigma Aldrich #T5580) were used at a concentration of 1 μM.

### Extracellular flux assays

HuT78 cells (scramble CTRL and KD) were plated in 96-well plates at a density of 0.5 or 1 × 10^6^ in 100 μl per well (total 3 × 10^6^ cells) and incubated for 22–24 h at 37 °C. The cells were harvested and washed with PBS and re-counted on a hemocytometer (INCYTO C-Chip). Half of the cells were re-suspended in the XF base media specific for the mitochondrial and the other half in XF base media specific for the glycolytic stress tests (below), respectively. For all extracellular flux assays, cells were plated on cell-tak-coated Seahorse XF96 cell culture microplates in (duplicate, triplicate or quadruplicate, depending on the cell count post-culture) at a density of 3–6 × 10^5^ cells per well. The assay plates were spin seeded for 5 min at 1000 rpm and incubated at 37 °C without CO_2_ prior to performing the assay on the Seahorse Bioscience XFe96 (Agilent). The mitochondrial stress test was performed in XF Base Media containing 10 mM glucose, 1 mM sodium pyruvate, and 2 mM l-glutamine, and the following inhibitors were added at the final concentrations: oligomycin (2 μM), carbonyl cyanide 4-(trifluoromethoxy)phenylhydrazone (FCCP) (2 μM), and rotenone/antimycin A (0.5 μM each). The glycolytic stress test was performed in XF Base Media containing 2 mM l-glutamine, and the following reagents were added at the final concentrations: glucose (10 mM), oligomycin (2 μM), and 2-deoxy-glycose (50 mM).

### shRNA-mediated knockdown of *APOBEC3G* in HuT78 cells

A3G knockdown in Hut78 cells was performed at the RPCCC gene modulation shared resource. For A3G knockdown, GIPZ human A3G shRNAs with the following clone ID’s were used: V2LHS_80856, V2LHS_80785, V2LHS_80786 (KD1a and KD1b), V3LHS_400156 (KD2), and V3LHS_303306 (Dharmacon). Lentiviruses were produced by cotransfection of 293T cells with A3G shRNA (or pGIPZ non-silencing control) along with psPAX2 and pMD2.G packaging plasmids, using the LipoD293 reagent (1:2.5 DNA to lipoD293 ratio) (SignaGen Laboratories) as per the manufacturer’s instructions. Culture supernatants were collected 48 and 72 h after transfection and cleared by filtration through 0.45 μm cellulose acetate syringe filter. For shRNA expression, 1 × 10^6^ Hut78 cells were pelleted and re-suspended with 1 ml culture supernatants containing the virus and 1 μl of 4 mg/ml polybrene. The cells were placed in 6-well plates and incubated for 30 min at 37 °C. The plate was sealed and spun at 1800 rpm for 45 min in a microtiter rotor (Beckman Coulter) at room temperature and then incubated for 6 h at 37 °C. After infection, the cells were centrifuged at 500*g* for 5 min and resuspended in IMDM media and incubated for 48 h at 37 °C. Puromycin (1 μg/ml) was added to the media to select for GFP-positive cells. Clone IDs V2LHS_80785 and V2LHS_80786 (KD1a) HuT78 cells were further sorted by the BD FACSaria II cell sorter (BD Biosciences) to obtain > 95% pure GFP-positive cells. A3G knockdown was verified by measuring the expression of *A3G* by qPCR and western blotting. Only two of five shRNA constructs [V2LHS_80786 (KD1a), V3LHS_400156 (KD2)] caused a significant reduction in A3G mRNA and protein expression and were used for further studies.

### RT-PCR and Sanger sequencing

Total RNA was isolated and reverse transcribed to generate cDNAs as described earlier [15]. DNA primers used for PCR were obtained from Integrated DNA Technologies and are noted in Additional file 10: Table S9. Primers used for PCR of cDNA templates were designed such that the amplicons spanned multiple exons. Agarose gel electrophoresis of PCR products was performed to confirm the generation of a single product in a PCR and then sequenced on the 3130 xL Genetic Analyzer (Life Technologies) at the RPCCC genomic core facility as described previously [17]. To quantify RNA editing level, the major and minor chromatogram peak heights at putative edited nucleotides were quantified with Sequencher 5.0/5.1 software (Gene Codes, MI). Since the software identifies a minor peak only if its height is at least 5% that of the major peak’s, we have considered 0.048 [=5/(100 + 5)] as the detection threshold [17, 27].

For quantitative PCR to assess *A3G*, *A3F*, and A3C gene expression, reactions using LightCycler™ 480 Probes Master and SYBR™ Green I dye were performed on a LightCycler™ 480 System (Roche). Quantification cycle (*C*_q_) values were calculated by the instrument software using the maximum second derivative method, and the mean *C*_q_ value of duplicate PCR reactions was used for analysis.

### Immunoblotting assays of cell lysates

Whole cell lysates were prepared and immunoblot was performed as described previously [15, 34]. APOBEC3G antiserum (Apo C17, catalog number 10082) was obtained from the NIH AIDS Reagent program [73, 74]; rabbit monoclonal phospho-eIF-2α (Ser 51) (product number-3398, DG98) was obtained from Cell Signaling Technology; mouse monoclonal anti-β-actin (product number AM4302, AC-15) was obtained from Life Technologies; mouse monoclonal anti-HIF1α (product number GTX628480, GT10211) and rabbit polyclonal anti-α-tubulin (product number GTX110432) were obtained from GeneTex and used at dilutions recommended by their manufacturers in 5% milk, except phospho-eiF-2α, which was diluted in 5% BSA. HRP-conjugated goat anti-mouse or anti-rabbit antibodies were purchased from Life Technologies and used at 1:2000 dilution followed by chemiluminescent detection of the proteins [15].

### Cell proliferation assay

Control and KD1a HuT78 cells (1 × 10^6^ cells in 100 μl per well) were seeded in 96-well round-bottom plates and incubated covered in the culture medium for 22 h in a 37 °C humidified hypoxia chamber (1% O_2_) or 37 °C humidified culture chamber (21% O_2_). Cellular dehydrogenase activity was determined using a WST-8 viability stain-based colorimetric assay (Dojindo Molecular Technologies, Inc.). Plates were read at 450 nm on an Epoch2 microplate reader (Biotek) using the Gen5 software (Biotek). Proliferation in KD2 and its control Hut78 cell line is measured by [^3^H]-thymidine (1 μCi per well) incorporation for 18 h with T cells in 96-well plate. After the initial cultures with cellular crowding (1 million cells per 0.1 ml volume in 96-well plate), 100,000 viable cells per well are used for the incorporation assay. Results are expressed as net counts per minute (cpm).

### Statistical analysis

Statistical analysis was performed using GraphPad Prism (7.03). A3G expression levels and mean editing levels in different cell types (Fig. 1) were first determined to be significantly statistically different by one-way ANOVA followed by the recommended multiple comparison tests. RNA editing level and cell proliferation differences between CTRL and KD Hut78 cells for each gene (Figs. 4 and 6) were examined by multiple *t* tests using the Holm-Sidak method, with alpha = 0.05. The effect of inhibitors on RNA editing was first determined to be statistically significant (Fig. 5) by two-way or one-way ANOVA followed by multiple comparisons of the treatment means for day 1 and/or day 2 using the recommended Dunnett’s multiple comparisons test. Respiration to glycolysis ratios (R/G) were calculated using basal respiration value for each well divided by the average glycolysis value of all wells for each experimental group (*n* = 3 for CTRL and KD HuT78 cells). These ratios were then normalized to the corresponding CTRL and KD T0 (unstressed cells) ratios within each experimental group, which are set to 1 (Fig. 6). The comparison of CTRL and KD HuT78 cells R/G ratios under stress, across all experiments, was performed by *t* test after normalizing the R/G values against the average of CTRL stress ratio in experiment 1. *p* values are indicated by stars: *p < 0.05, **p < 0.01, ***p < 0.001, and ****p < 0.0001. Fisher’s exact and chi-squared tests are performed online at http://www.quantitativeskills.com/sisa/. All reported *p* values are two-sided.

### Others

Gene expression meta-analysis of A3G is performed on two online platforms: (1) BIOGPS [75], searchable collection of thousands of gene expression datasets, and (2) Cancer Cell Line Encyclopedia (CCLE) portal [35]. CCLE database contains over 1000 cell lines. Weblogo is created at http://weblogo.berkeley.edu/ with default parameters [76].

## Additional files

Additional file 1: **Figure S1-S12.** Supplementary figures. (PPTX 1499 kb)

Additional file 2: **Table S1.** C>U RNA editing events after the first filter (FDR < 0.05). These events were present at least 5% frequency in any sample and overrepresented in the hypoxia or normoxia group. (XLSX 286 kb)

Additional file 3: **Table S2.** C>U RNA editing events after the second filter (− 1 C or T). These events have C or T at − 1 nucleotide position. (XLSX 222 kb)

Additional file 4: **Table S3.** C>U RNA editing events after the third filter (stem-loop). These events are located at the 3′-end of loops in putative stem-loop structures within exons or UTRs. (XLSX 59 kb)

Additional file 5: **Table S4.** C>U RNA editing events shared between NK cells and 293T/APOBEC3G overexpression system. (XLSX 22 kb)

Additional file 6: **Table S5.** C>U RNA editing events shared between NK cells and 293T/APOBEC3A, and NK cells, 293T/APOBEC3A and 293T/APOBEC3G overexpression systems. (XLSX 14 kb)

Additional file 7: **Table S6.** A>I RNA editing events in RADAR database that are induced by hypoxia in NK cells. (XLSX 11 kb)

Additional file 8: **Table S7.** Evolutionary conservation analysis of all non-synonymous C>U RNA editing sites. (XLSX 18 kb)

Additional file 9: **Table S8.** Gene expression levels in normoxic and hypoxic NK cells. (XLSX 3002 kb)

Additional file 10: **Table S9.** Oligonucleotide primer sequences used for PCR amplification and Sanger sequencing. (XLSX 10 kb)
