# Supplementary material for: Mitochondrial hypoxic stress induces widespread RNA editing by APOBEC3G in natural killer cells
Source: Genome Biol. 2019 Feb 21;20:37. doi: 10.1186/s13059-019-1651-1 (PMC6383285; doi:10.1186/s13059-019-1651-1)
Supplement: Supplementary file 1 — Figure S1-S12. Supplementary figures. (PPTX 1499 kb) [file 13059_2019_1651_MOESM1_ESM.pptx]

## Slide 1
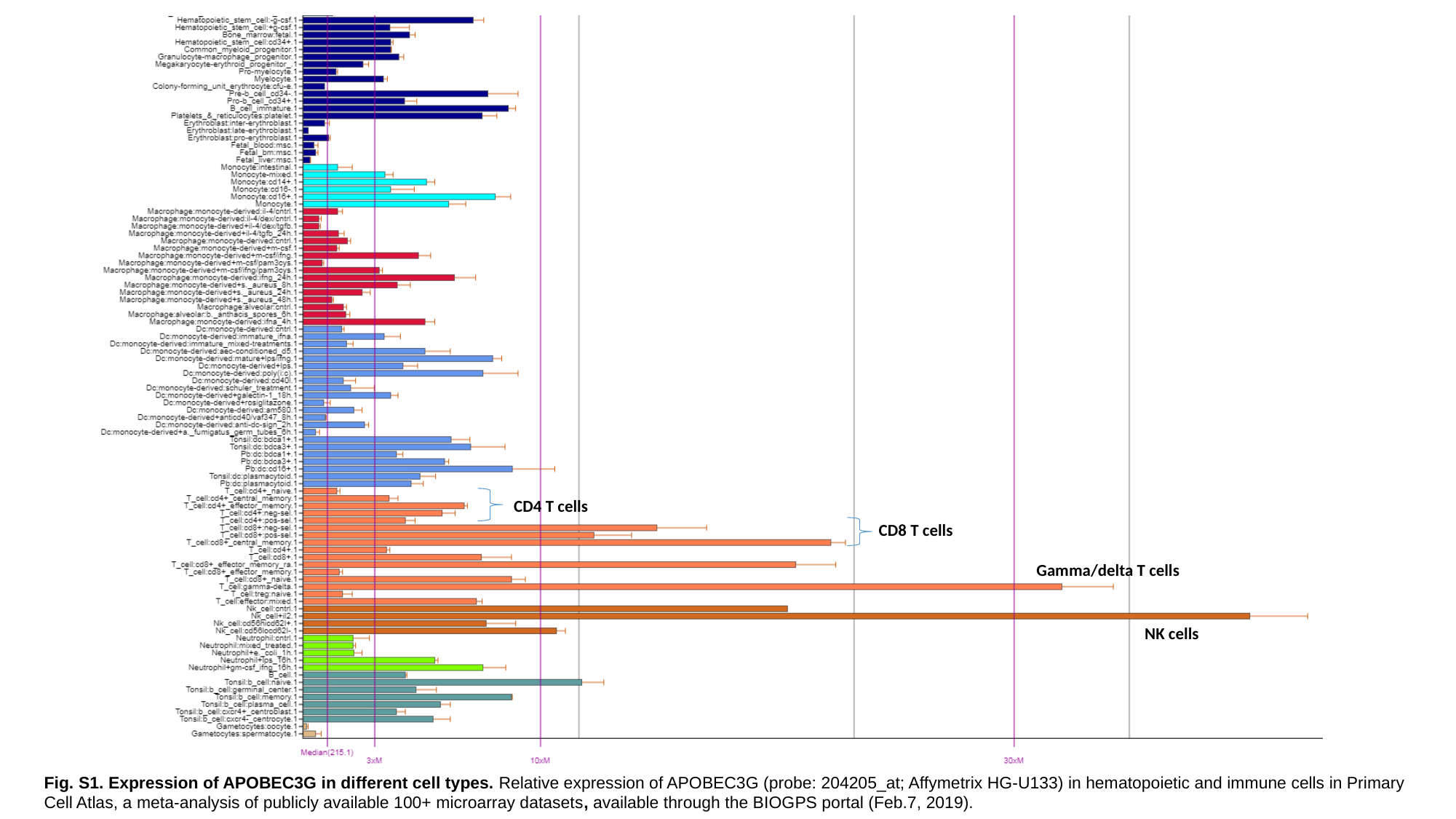

CD4 T cells
CD8 T cells
Gamma/delta T cells
NK cells
Fig. S1. Expression of APOBEC3G in different cell types. Relative expression of APOBEC3G (probe: 204205_at; Affymetrix HG-U133) in hematopoietic and immune cells in Primary Cell Atlas, a meta-analysis of publicly available 100+ microarray datasets, available through the BIOGPS portal (Feb.7, 2019).

## Slide 2
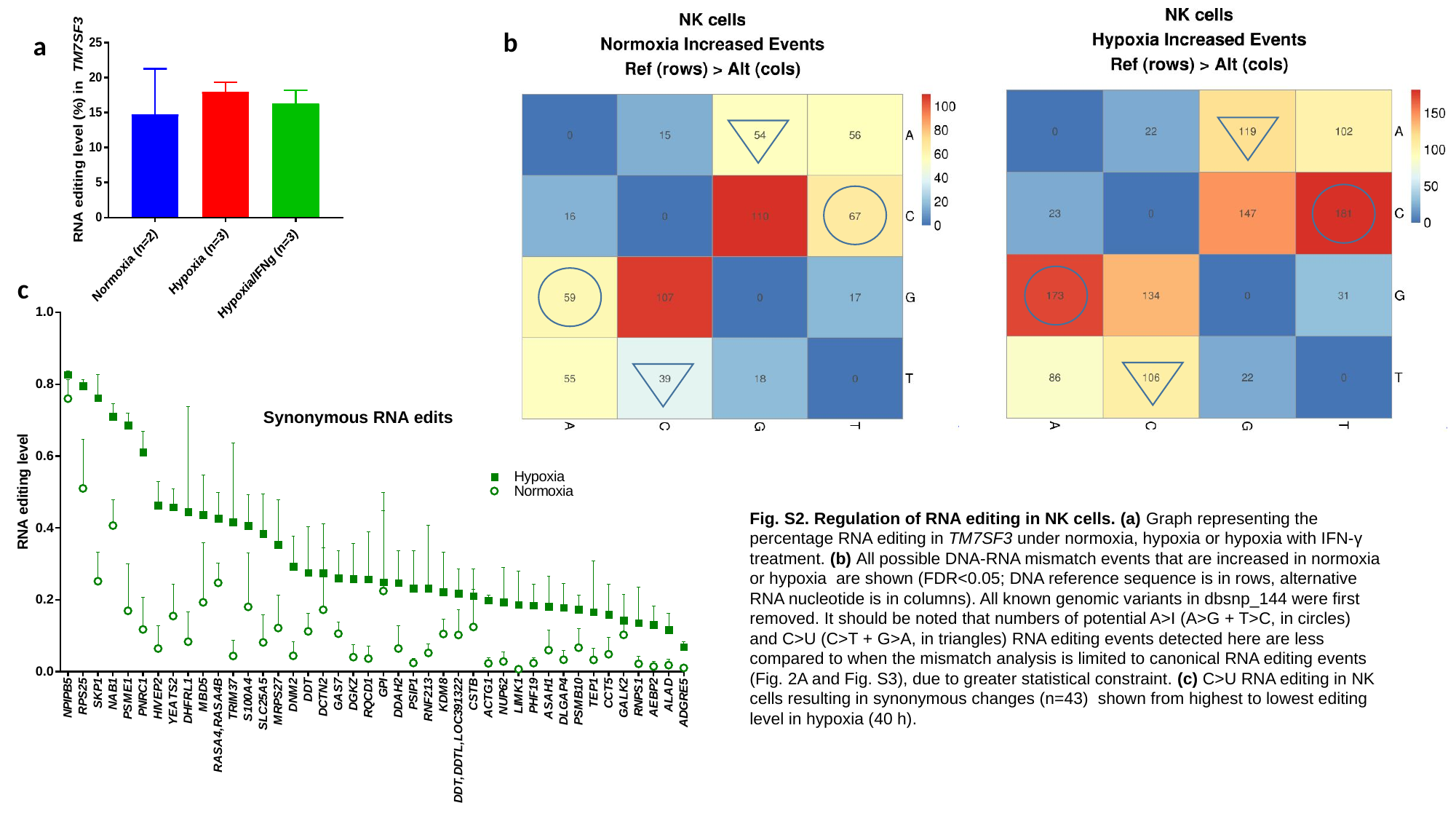

b
a
c
Fig. S2. Regulation of RNA editing in NK cells. (a) Graph representing the percentage RNA editing in TM7SF3 under normoxia, hypoxia or hypoxia with IFN-γ treatment. (b) All possible DNA-RNA mismatch events that are increased in normoxia or hypoxia are shown (FDR<0.05; DNA reference sequence is in rows, alternative RNA nucleotide is in columns). All known genomic variants in dbsnp_144 were first removed. It should be noted that numbers of potential A>I (A>G + T>C, in circles) and C>U (C>T + G>A, in triangles) RNA editing events detected here are less compared to when the mismatch analysis is limited to canonical RNA editing events (Fig. 2A and Fig. S3), due to greater statistical constraint. (c) C>U RNA editing in NK cells resulting in synonymous changes (n=43) shown from highest to lowest editing level in hypoxia (40 h).

## Slide 3
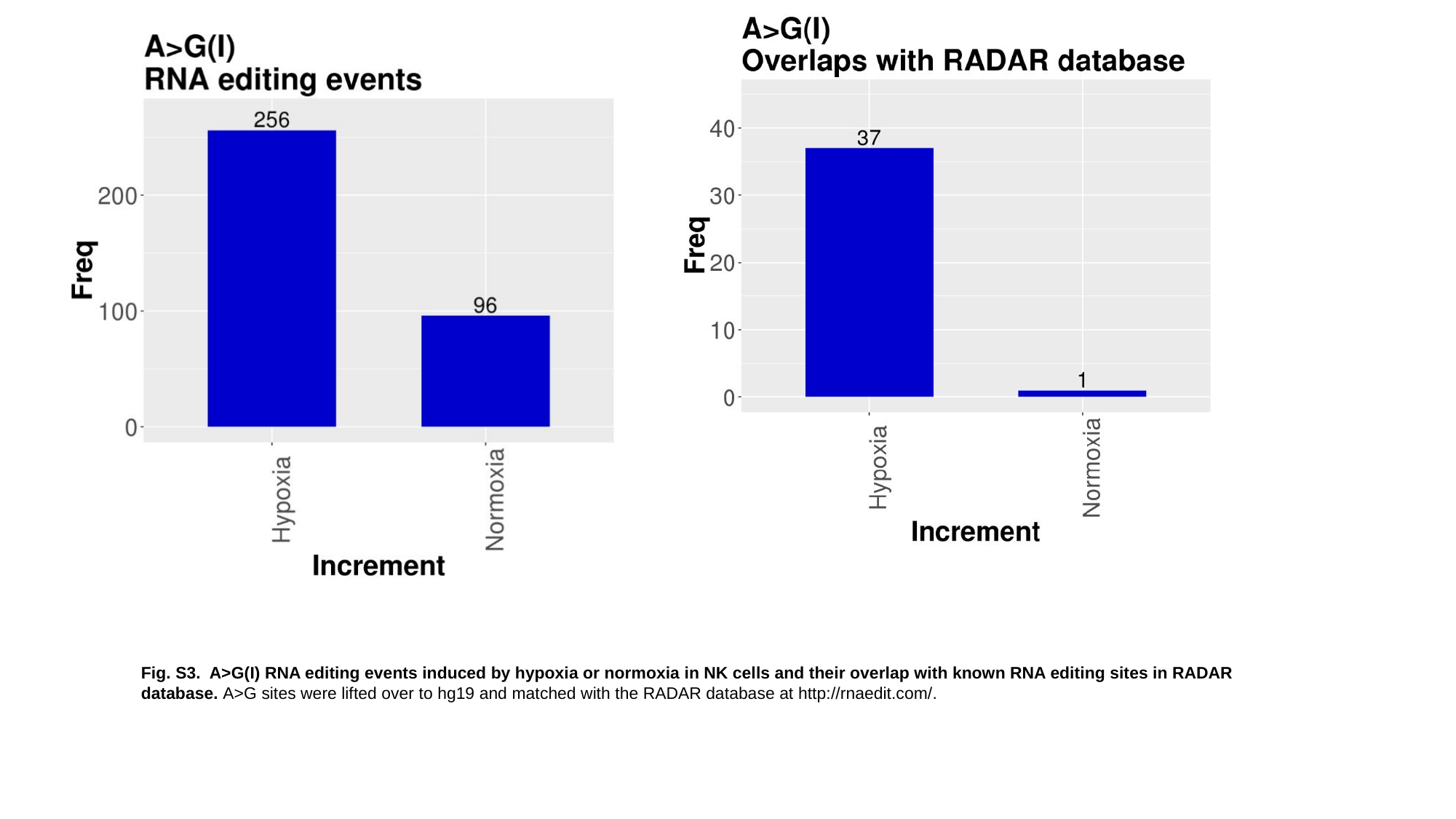

Fig. S3. A>G(I) RNA editing events induced by hypoxia or normoxia in NK cells and their overlap with known RNA editing sites in RADAR database. A>G sites were lifted over to hg19 and matched with the RADAR database at http://rnaedit.com/.

## Slide 4
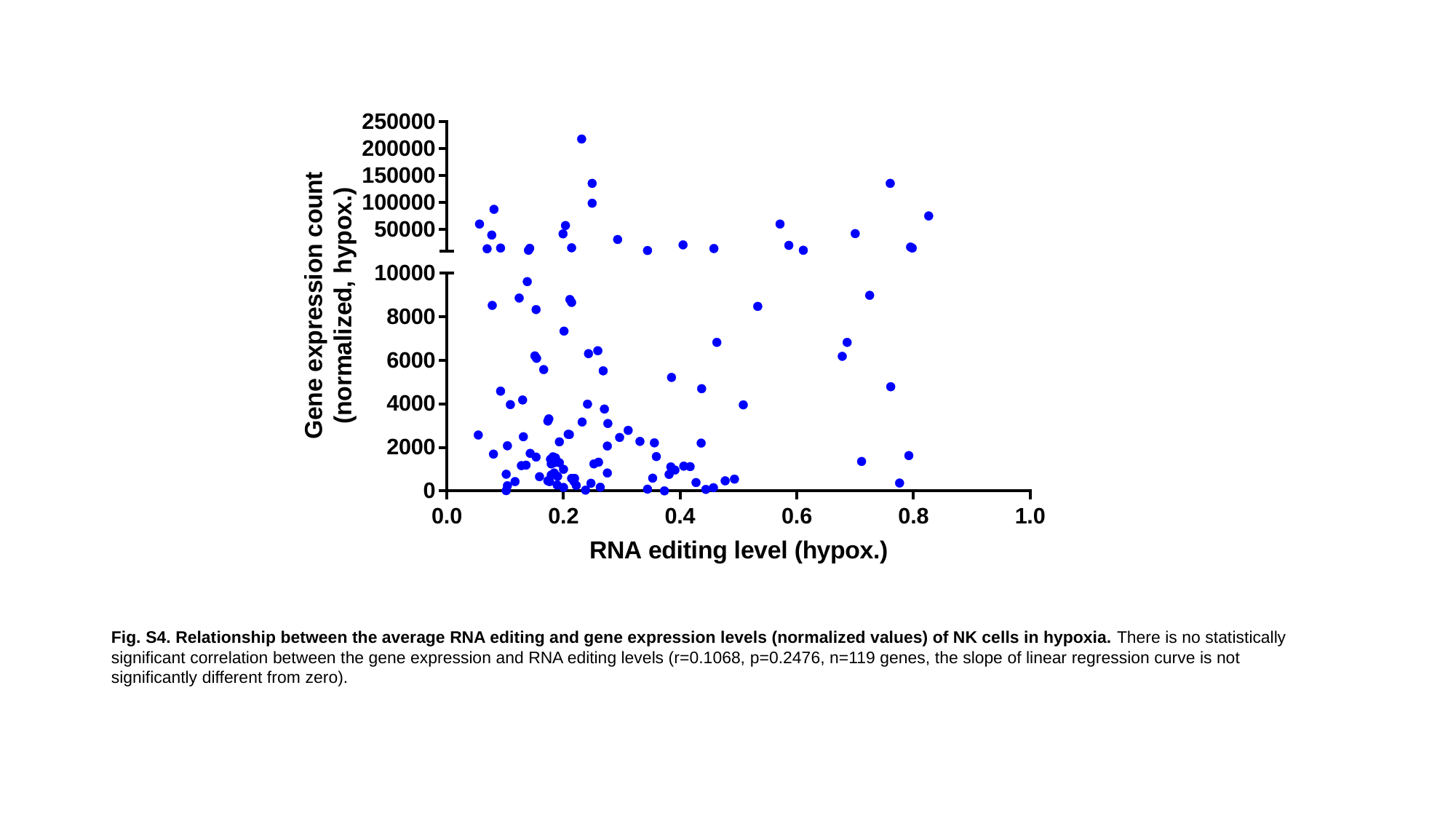

Fig. S4. Relationship between the average RNA editing and gene expression levels (normalized values) of NK cells in hypoxia. There is no statistically significant correlation between the gene expression and RNA editing levels (r=0.1068, p=0.2476, n=119 genes, the slope of linear regression curve is not significantly different from zero).

## Slide 5
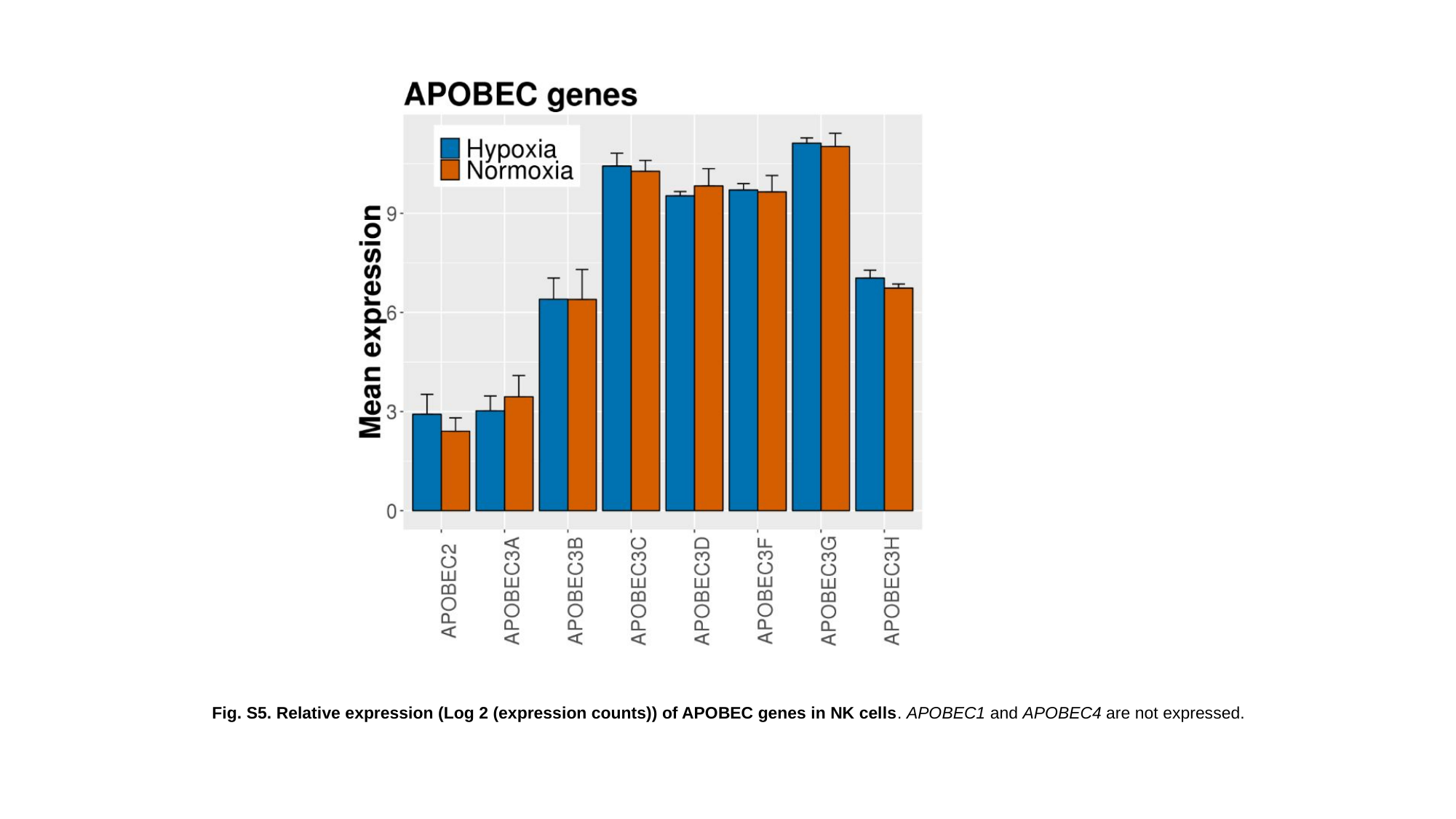

Fig. S5. Relative expression (Log 2 (expression counts)) of APOBEC genes in NK cells. APOBEC1 and APOBEC4 are not expressed.

## Slide 6
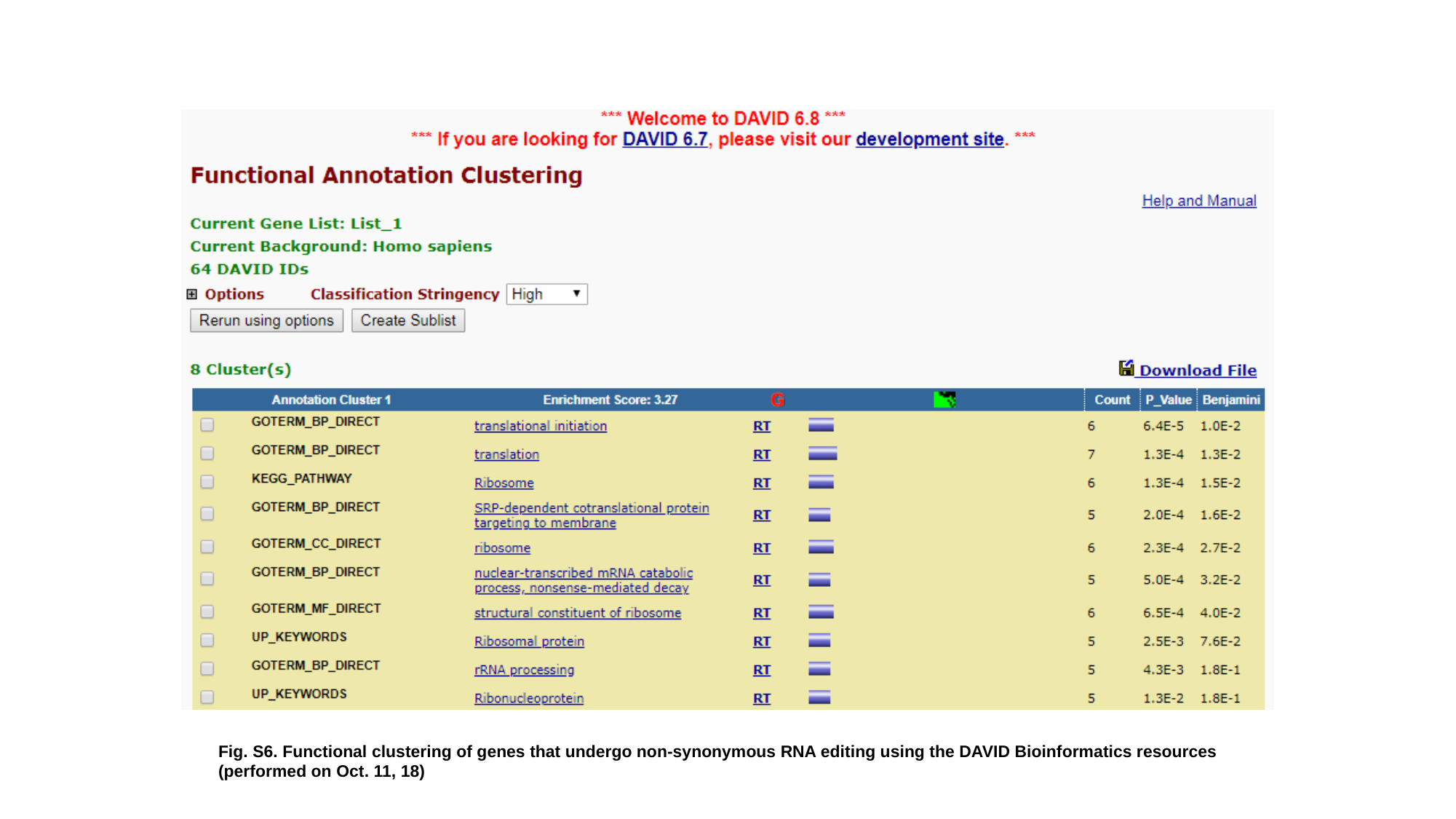

Fig. S6. Functional clustering of genes that undergo non-synonymous RNA editing using the DAVID Bioinformatics resources (performed on Oct. 11, 18)

## Slide 7
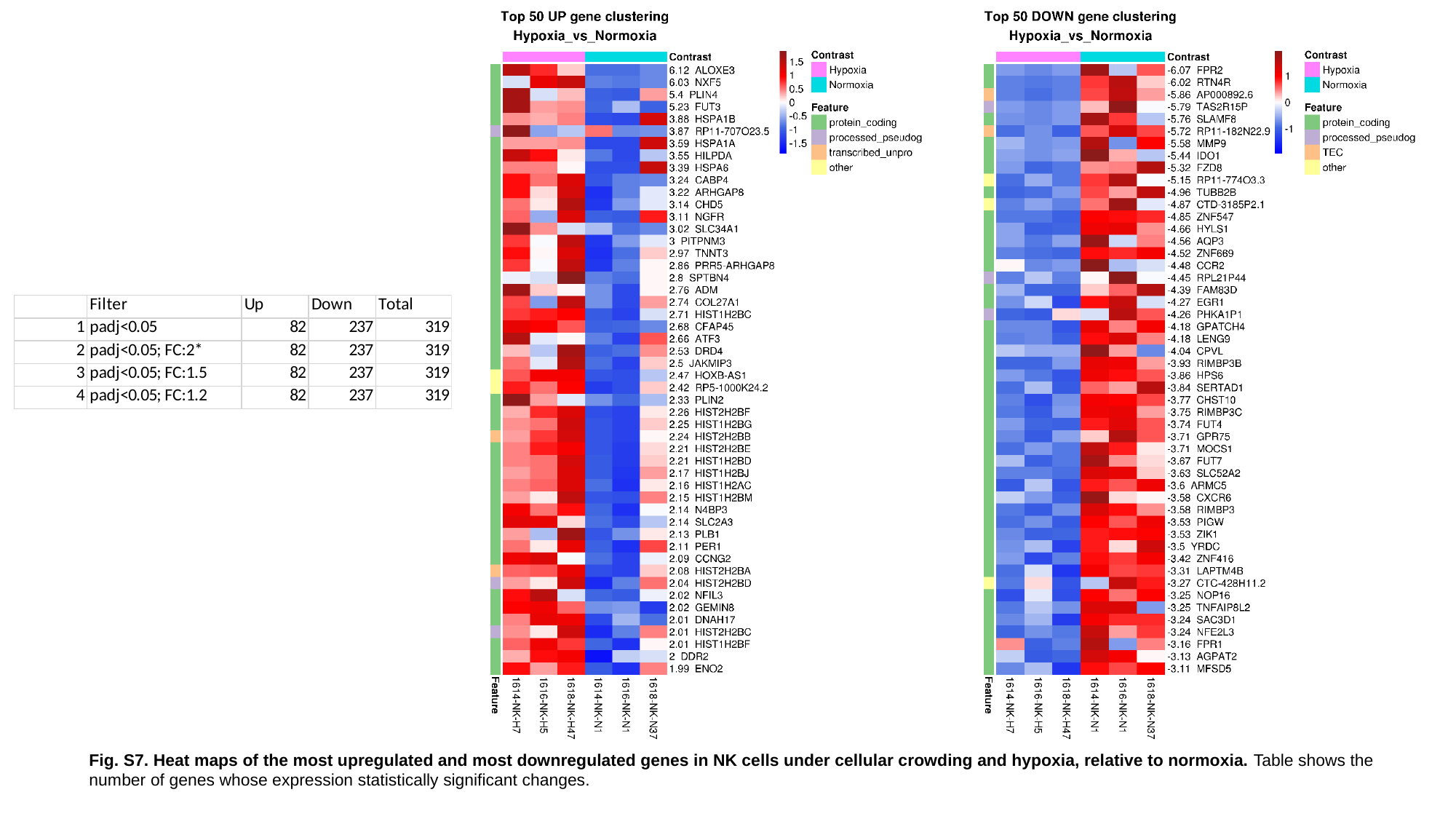

Fig. S7. Heat maps of the most upregulated and most downregulated genes in NK cells under cellular crowding and hypoxia, relative to normoxia. Table shows the number of genes whose expression statistically significant changes.

## Slide 8
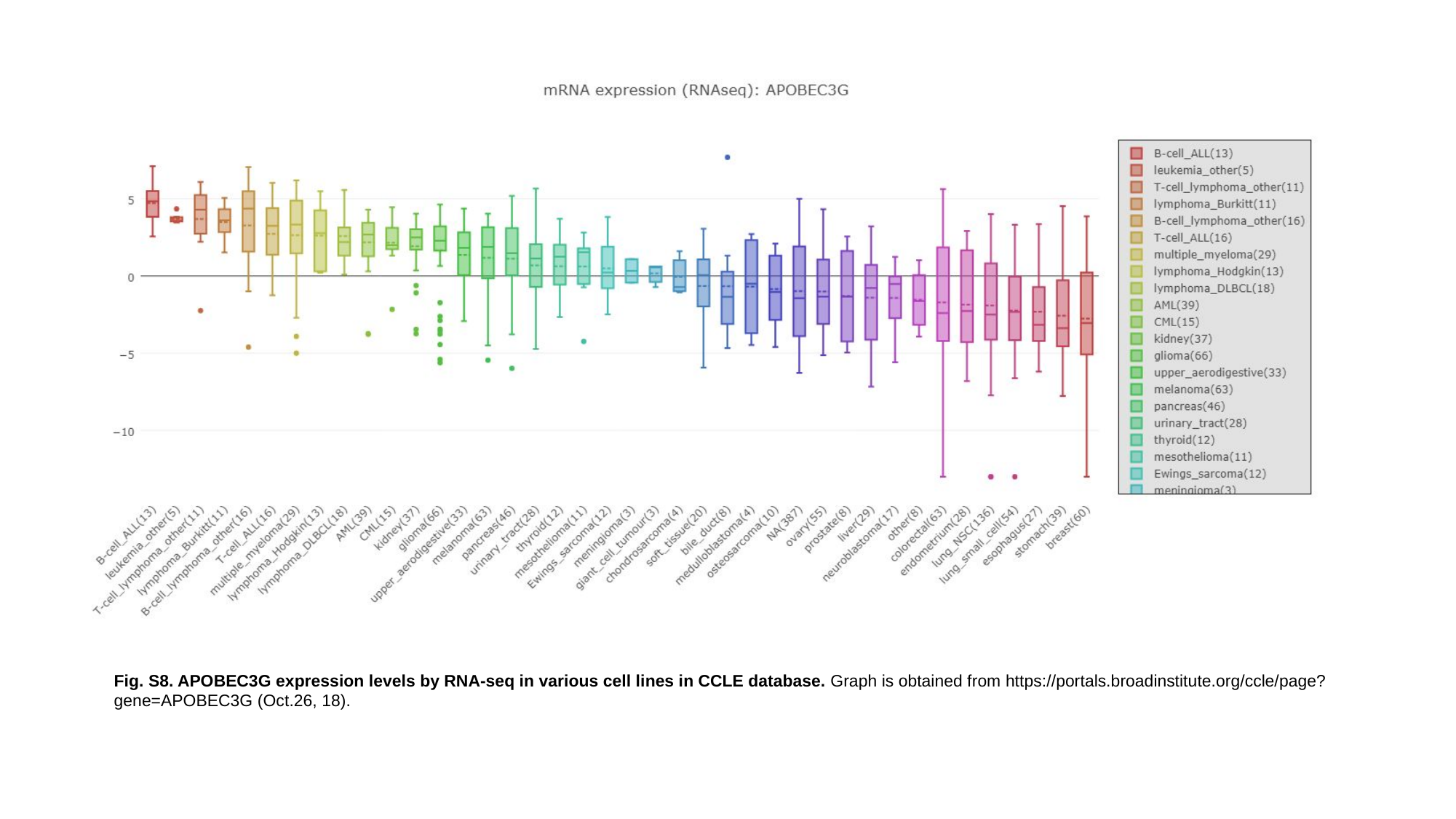

Fig. S8. APOBEC3G expression levels by RNA-seq in various cell lines in CCLE database. Graph is obtained from https://portals.broadinstitute.org/ccle/page?gene=APOBEC3G (Oct.26, 18).

## Slide 9
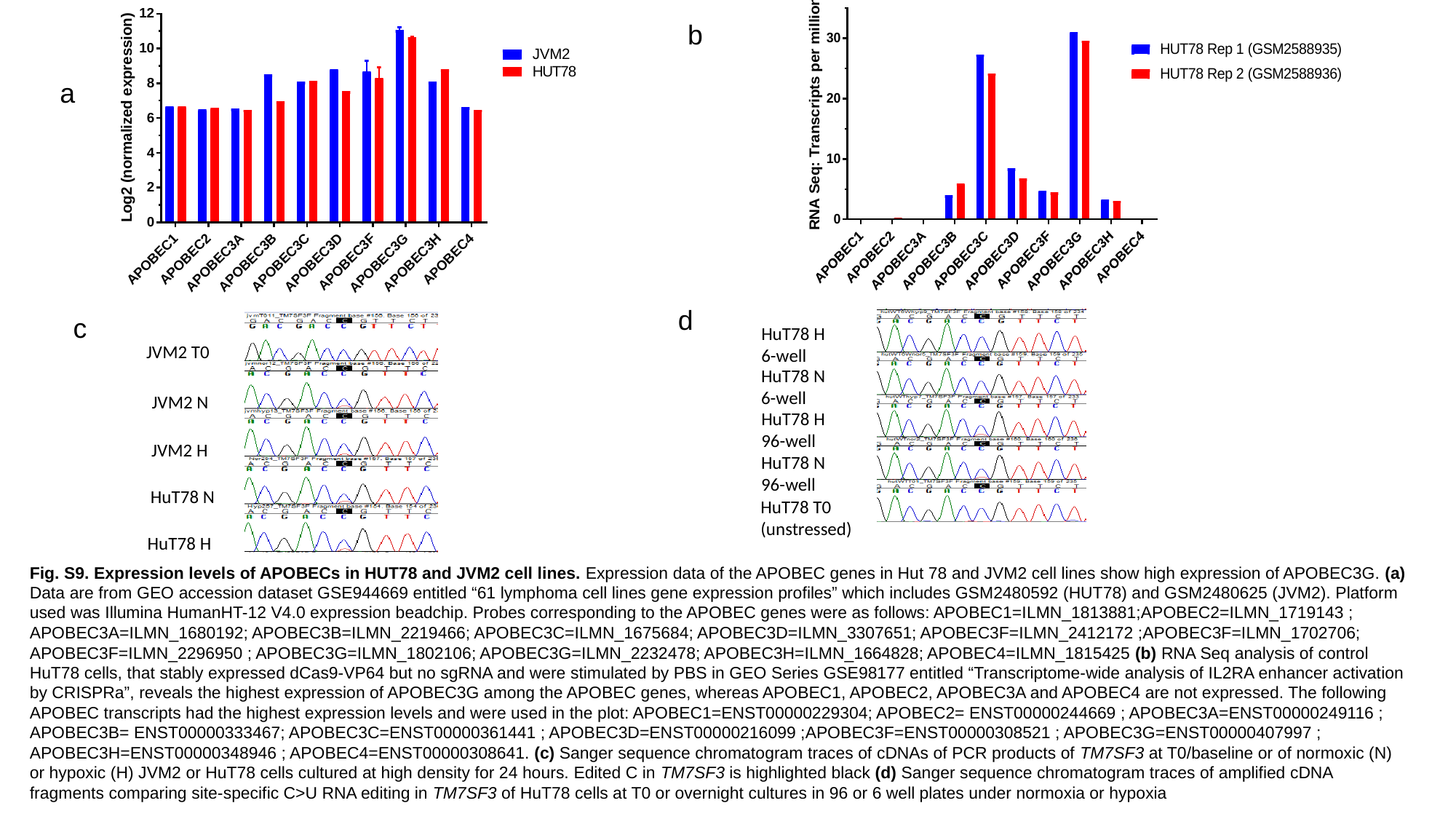

b
a
d
c
HuT78 H
6-well
HuT78 N
6-well
HuT78 H
96-well
HuT78 N
96-well
HuT78 T0
(unstressed)
JVM2 T0
JVM2 N
JVM2 H
HuT78 N
HuT78 H
Fig. S9. Expression levels of APOBECs in HUT78 and JVM2 cell lines. Expression data of the APOBEC genes in Hut 78 and JVM2 cell lines show high expression of APOBEC3G. (a) Data are from GEO accession dataset GSE944669 entitled “61 lymphoma cell lines gene expression profiles” which includes GSM2480592 (HUT78) and GSM2480625 (JVM2). Platform used was Illumina HumanHT-12 V4.0 expression beadchip. Probes corresponding to the APOBEC genes were as follows: APOBEC1=ILMN_1813881;APOBEC2=ILMN_1719143 ; APOBEC3A=ILMN_1680192; APOBEC3B=ILMN_2219466; APOBEC3C=ILMN_1675684; APOBEC3D=ILMN_3307651; APOBEC3F=ILMN_2412172 ;APOBEC3F=ILMN_1702706; APOBEC3F=ILMN_2296950 ; APOBEC3G=ILMN_1802106; APOBEC3G=ILMN_2232478; APOBEC3H=ILMN_1664828; APOBEC4=ILMN_1815425 (b) RNA Seq analysis of control HuT78 cells, that stably expressed dCas9-VP64 but no sgRNA and were stimulated by PBS in GEO Series GSE98177 entitled “Transcriptome-wide analysis of IL2RA enhancer activation by CRISPRa”, reveals the highest expression of APOBEC3G among the APOBEC genes, whereas APOBEC1, APOBEC2, APOBEC3A and APOBEC4 are not expressed. The following APOBEC transcripts had the highest expression levels and were used in the plot: APOBEC1=ENST00000229304; APOBEC2= ENST00000244669 ; APOBEC3A=ENST00000249116 ; APOBEC3B= ENST00000333467; APOBEC3C=ENST00000361441 ; APOBEC3D=ENST00000216099 ;APOBEC3F=ENST00000308521 ; APOBEC3G=ENST00000407997 ; APOBEC3H=ENST00000348946 ; APOBEC4=ENST00000308641. (c) Sanger sequence chromatogram traces of cDNAs of PCR products of TM7SF3 at T0/baseline or of normoxic (N) or hypoxic (H) JVM2 or HuT78 cells cultured at high density for 24 hours. Edited C in TM7SF3 is highlighted black (d) Sanger sequence chromatogram traces of amplified cDNA fragments comparing site-specific C>U RNA editing in TM7SF3 of HuT78 cells at T0 or overnight cultures in 96 or 6 well plates under normoxia or hypoxia

## Slide 10
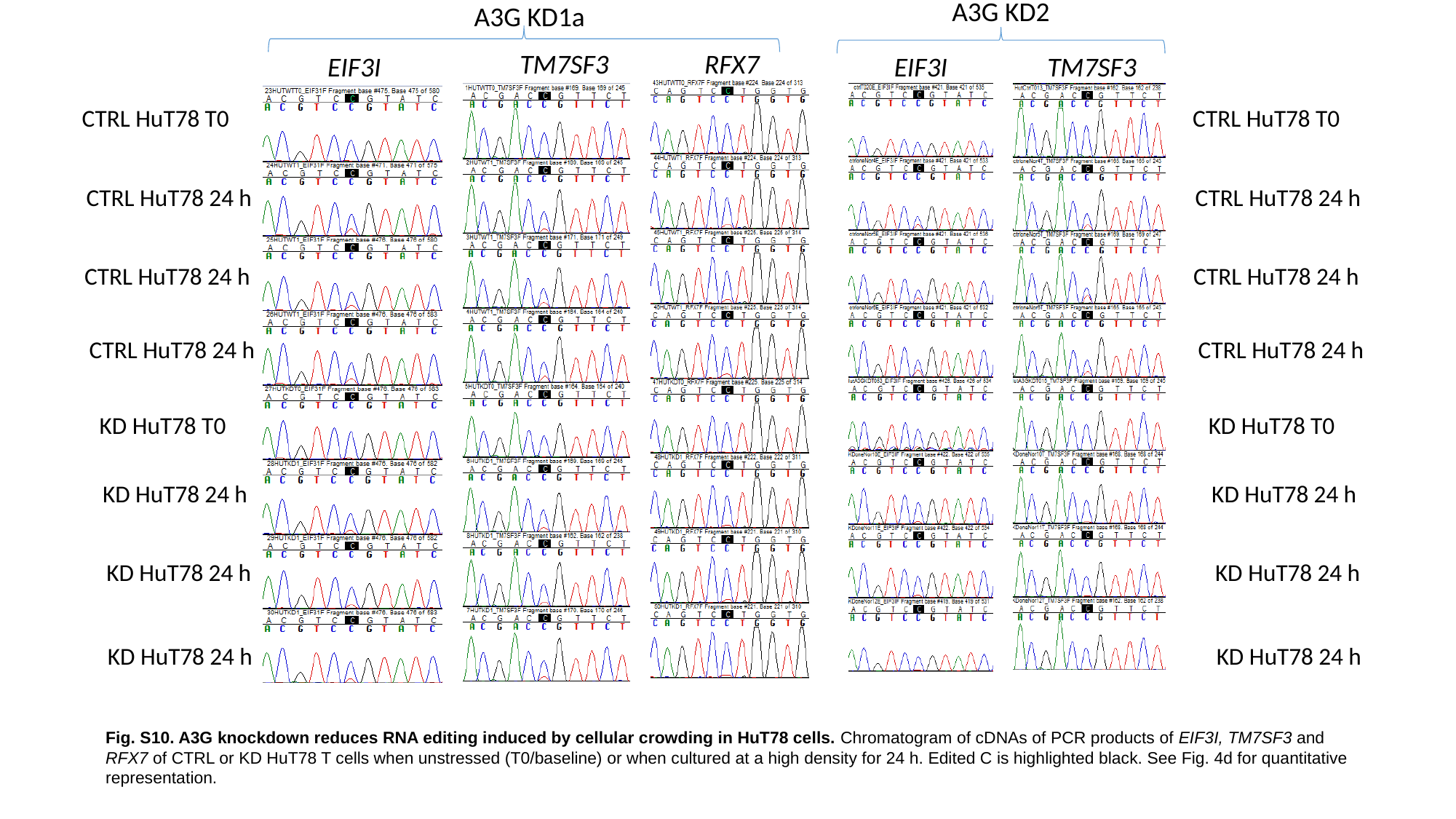

A3G KD2
A3G KD1a
TM7SF3
RFX7
EIF3I
EIF3I
TM7SF3
CTRL HuT78 T0
CTRL HuT78 T0
CTRL HuT78 24 h
CTRL HuT78 24 h
CTRL HuT78 24 h
CTRL HuT78 24 h
CTRL HuT78 24 h
CTRL HuT78 24 h
KD HuT78 T0
KD HuT78 T0
KD HuT78 24 h
KD HuT78 24 h
KD HuT78 24 h
KD HuT78 24 h
KD HuT78 24 h
KD HuT78 24 h
Fig. S10. A3G knockdown reduces RNA editing induced by cellular crowding in HuT78 cells. Chromatogram of cDNAs of PCR products of EIF3I, TM7SF3 and RFX7 of CTRL or KD HuT78 T cells when unstressed (T0/baseline) or when cultured at a high density for 24 h. Edited C is highlighted black. See Fig. 4d for quantitative representation.

## Slide 11
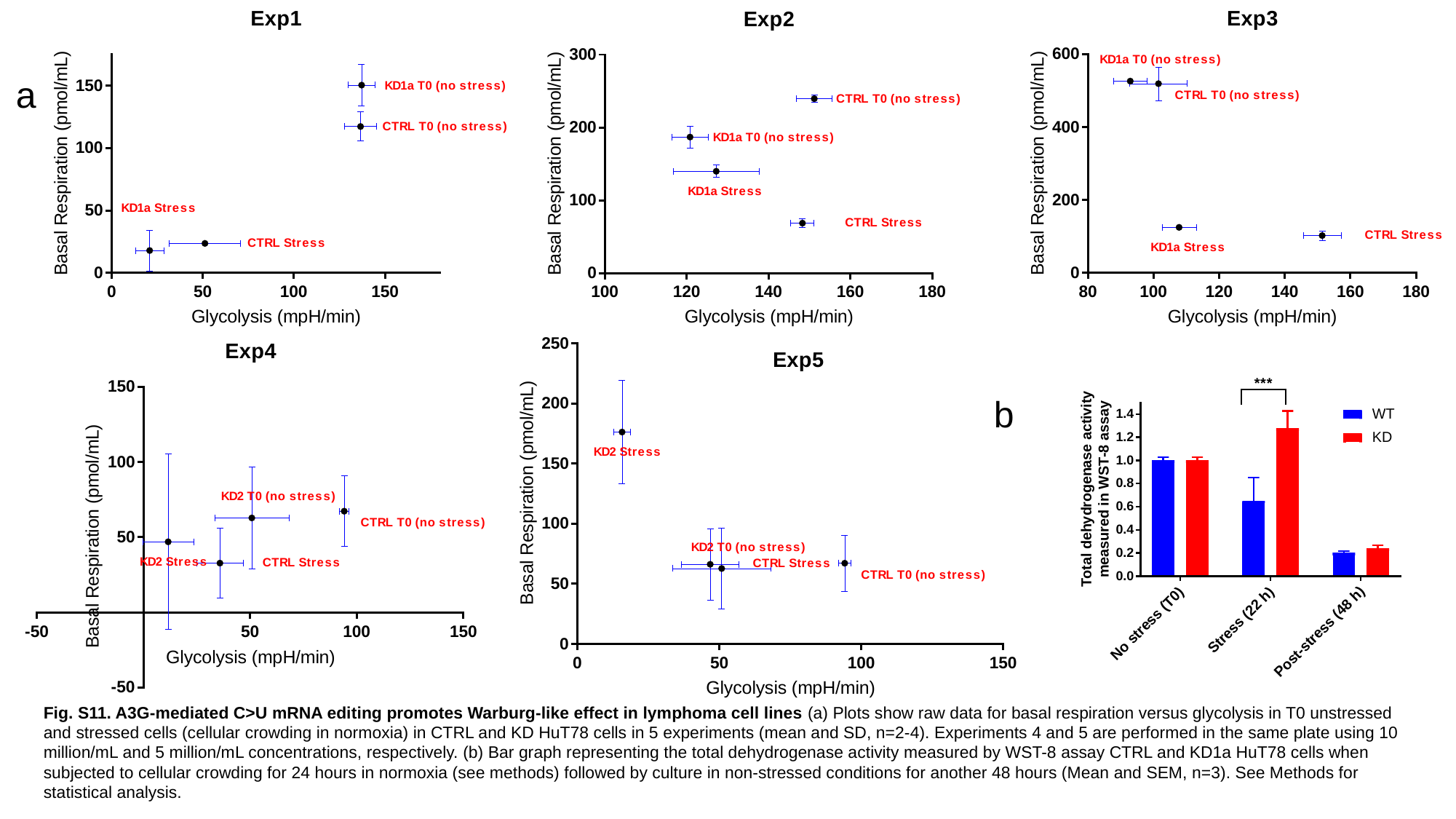

a
b
Fig. S11. A3G-mediated C>U mRNA editing promotes Warburg-like effect in lymphoma cell lines (a) Plots show raw data for basal respiration versus glycolysis in T0 unstressed and stressed cells (cellular crowding in normoxia) in CTRL and KD HuT78 cells in 5 experiments (mean and SD, n=2-4). Experiments 4 and 5 are performed in the same plate using 10 million/mL and 5 million/mL concentrations, respectively. (b) Bar graph representing the total dehydrogenase activity measured by WST-8 assay CTRL and KD1a HuT78 cells when subjected to cellular crowding for 24 hours in normoxia (see methods) followed by culture in non-stressed conditions for another 48 hours (Mean and SEM, n=3). See Methods for statistical analysis.

## Slide 12
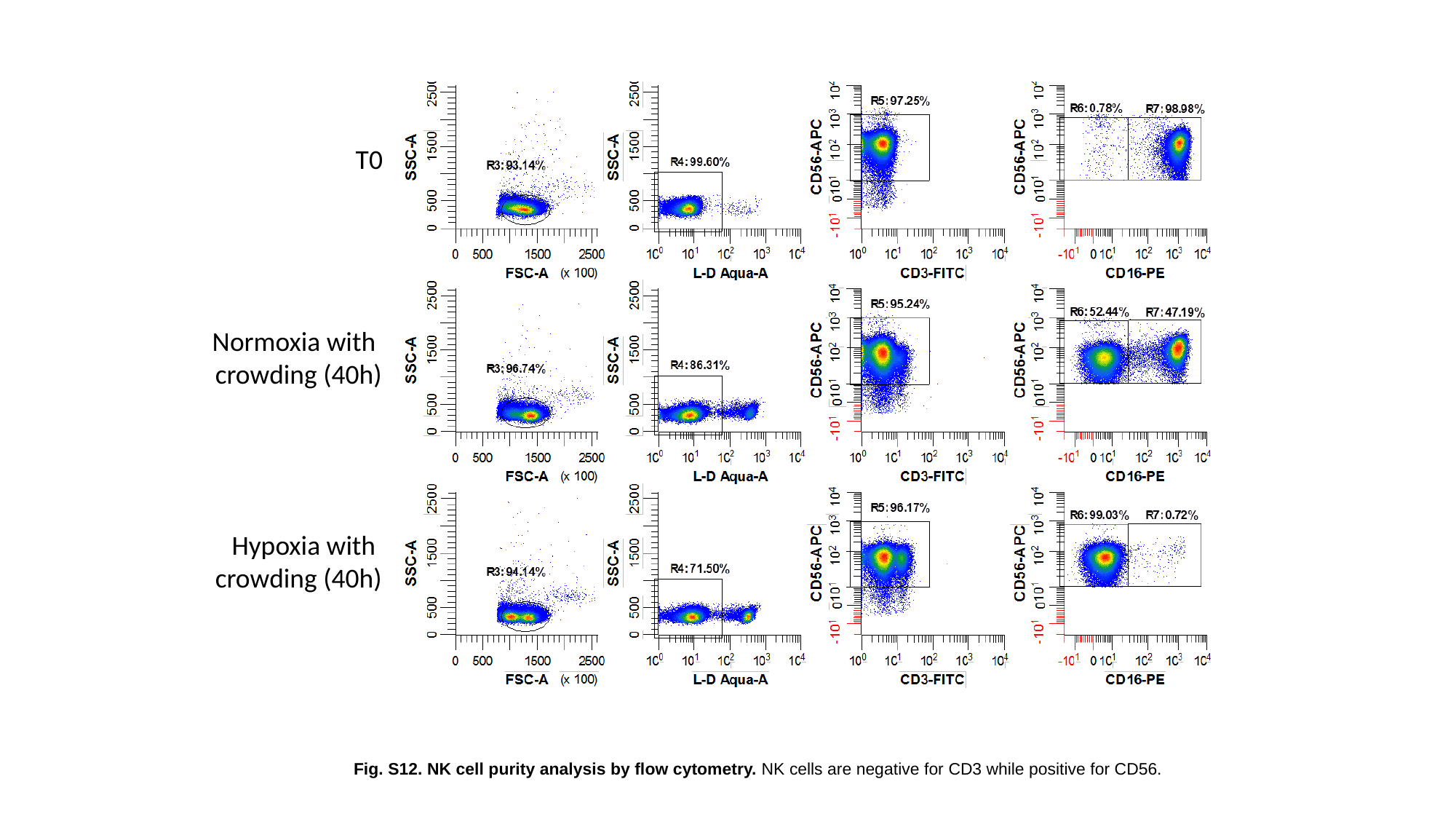

T0
Normoxia with
crowding (40h)
Hypoxia with
crowding (40h)
Fig. S12. NK cell purity analysis by flow cytometry. NK cells are negative for CD3 while positive for CD56.
